# Functional analysis of LHCSR1, a protein catalyzing NPQ in mosses, by heterologous expression in *Arabidopsis thaliana*

**DOI:** 10.1007/s11120-019-00656-3

**Published:** 2019-07-03

**Authors:** Ioannis Dikaios, Christo Schiphorst, Luca Dall’Osto, Alessandro Alboresi, Roberto Bassi, Alberta Pinnola

**Affiliations:** 1 Department of Biotechnology, University of Verona, Verona, 37134 Italy; 2 Department of Biology and Biotechnology, University of Pavia, Pavia, 27100 Italy

**Keywords:** LHCSR, NPQ, Photoprotection, Heterologous expression, *P. patens*, *A. thaliana*

## Abstract

Non-photochemical quenching, NPQ, of chlorophyll fluorescence regulates the heat dissipation of chlorophyll excited states and determines the efficiency of the oxygenic photosynthetic systems. NPQ is regulated by a pH-sensing protein, responding to the chloroplast lumen acidification induced by excess light, coupled to an actuator, a chlorophyll/xanthophyll subunit where quenching reactions are catalyzed. In plants, the sensor is PSBS, while the two pigment-binding proteins Lhcb4 (also known as CP29) and LHCII are the actuators. In algae and mosses, stress-related light-harvesting proteins (LHCSR) comprise both functions of sensor and actuator within a single subunit. Here, we report on expressing the *lhcsr1* gene from the moss *Physcomitrella patens* into several *Arabidopsis thaliana npq4* mutants lacking the pH sensing PSBS protein essential for NPQ activity. The heterologous protein LHCSR1 accumulates in thylakoids of *A. thaliana* and NPQ activity can be partially restored. Complementation of double mutants lacking, besides PSBS, specific xanthophylls, allowed analyzing chromophore requirement for LHCSR-dependent quenching activity. We show that the partial recovery of NPQ is mostly due to the lower levels of Zeaxanthin in *A. thaliana* in comparison to *P. patens*. Complemented *npq2npq4* mutants, lacking besides PSBS, Zeaxanthin Epoxidase, showed an NPQ recovery of up to 70% in comparison to *A. thaliana* wild type. Furthermore, we show that Lutein is not essential for the folding nor for the quenching activity of LHCSR1. In short, we have developed a system to study the function of LHCSR proteins using heterologous expression in a variety of *A. thaliana* mutants.

**Electronic supplementary material:**

The online version of this article (10.1007/s11120-019-00656-3) contains supplementary material, which is available to authorized users.

## Introduction

The need for a balance between light harvesting and photoprotection is one of the key driving forces that shaped adaptation of photosynthetic eukaryotic organisms on Earth (Genty et al. 1990; Müller et al. 2001; Baker 2008). Non-photochemical quenching (NPQ) of chlorophyll (Chl) fluorescence acts through modulating the dissipation of Chl excited states into heat and balances the efficiency of the photosynthetic systems versus the electron transport rate, thus avoiding photo-oxidative stress and photoinhibition due to excess light. NPQ includes components with different induction and relaxation kinetics: the fastest (1–2 min) and rapidly reversible type, qE, depends on a trans-thylakoid ΔpH promoted by excess light (Horton et al. 1996; Kramer et al. 1999; Kanazawa and Kramer 2002) which protonates specific residues on pH sensitive trigger proteins (Li et al. 2004; Ballottari et al. 2016); qZ, is activated in 8–10 min and also depends on low luminal pH through the activation of violaxanthin (Vio) de-epoxidase (VDE), a lumenal enzyme-converting zeaxanthin (Zea) from pre-existing Vio. The slowest component, called qI, for photoInhibitory quenching, comprises components from the slow and reversible inactivation of Photosystem II (PSII) reaction centers as well as other long-term processes involved in acclimation to the light environment (Brooks et al. 2013). In some organisms, such as *Chlamydomonas reinhardtii* an additional component, qT, is due to the displacement of LHCII from PSII to PSI upon phosphorylation (Allorent et al. 2013). qE activation depends on a sensor for lumenal pH, induced by excess light, coupled to a Chl/xanthophyll actuator subunit where quenching reactions are catalyzed upon the establishment of specific pigment–pigment interactions (Allorent et al. 2013). The protein PSBS is a typical pH sensor (Li et al. 2000) which does not bind chromophores (Dominici et al. 2002; Fan et al. 2015), but is able to activate quenching within the interacting antenna protein subunits Lhcb4 (CP29) (Ahn et al. 2008; de Bianchi et al. 2011) and LHCII (Ruban et al. 2007; Dall’Osto et al. 2017). In the case of algae the trigger of qE is LHCSR, a pigment binding protein (Peers et al. 2009) which also hosts protonatable residues (Liguori et al. 2013; Ballottari et al. 2016) thus comprising both sensing and catalytic functions in a single subunit (Bonente et al. 2011). The moss *Physcomitrella patens*, a descendant from an evolutionary intermediate between algae and plants, hosts both PSBS and LHCSR each active in qE (Alboresi et al. 2010; Gerotto et al. 2012) which suggests that LHCSR might be active in vascular plants. Modification of qE, timescales in which PSBS and LHCSR1 are active, can improve crop productivity depending on the growth conditions (Horton 2000). Since the NPQ activity of PSBS and LHCSR1 are cumulative in *P. patens* and LHCSR1 is not present in plants, re-introducing LHCSR1 in vascular plants could enhance the dynamic range of NPQ with positive effects on crop productivity. In this work, we used the *npq4* mutant of *A. thaliana,* lacking PSBS and, therefore qE, as a host for the expression of *P. patens* LHCSR1. We proceeded to verify the possibility of expressing LHCSR1 in vascular plants and its capability to complement the NPQ function in genotypes lacking PSBS. The availability of a large library of *A. thaliana* mutants affected in energy dissipation makes transformation by *Agrobacterium* mediated floral dipping (Clough and Bent 1998), an efficient tool for elucidation of the quenching mechanism in LHCSR1. As a proof of concept, we determined the requirement of specific xanthophyll co-factors for LHCSR1-dependent quenching.

## Materials and methods

### Cloning of LHCSR1 cDNA, *A. thaliana* transformation and screening

The fragment corresponding to *LHCSR1* (Locus XM_024529130) was amplified from *P. patens* total cDNA obtained from 6-days-old plants grown on minimal medium, RNA was isolated using TRI Reagent^®^ Protocol (T9424, Sigma-Aldrich) and cDNA was synthetized using M-MLV Reverse Transcriptase (M1302, Sigma-Aldrich) and Oligo(dT)_23_ (O4387, Sigma-Aldrich). Primers including *attB* sequences for the gateway technology (Invitrogen™) were designed to anneal 27 base pairs upstream of the ATG codon (*Pp*LHCSR1attB1 5′-GGGGACAAGTTTGTACAAAAAAGCAGGCTCCAATCTCGAGCTTTTGCT-3′) and 107 base pairs downstream of the stop codon (*Pp*LHCSR1attB 5′- GGGGACCACTTTGTACAAGAAAGCTGGGTCGACTGCGAATCAATCAGAA-3′). The PCR-product was first cloned in pDONR™221 Vector (12536-017, Invitrogen™) and then recombined into the pH7WG2 binary vector (Karimi et al. 2002) to make the *35S*::*lhcsr1* construct. The accuracy of the cloning was verified by DNA digestion and sequencing and the plasmid was transferred to *Agrobacterium tumefaciens* strain GV3101 (Zhang et al. 2006). *A. thaliana* plants were transformed by the floral dip method and transgenic plants were selected on Murashige-Skoog medium supplemented by hygromycin (25 mg L^−1^) and carbenicillin (100 mg L^−1^) (Clough and Bent 1998).

### Plant material and growth conditions

*Physcomitrella patens* protonema tissue was grown in petri dishes containing minimum PPNO_3_ medium (Ashton et al. 1979) enriched with 0.5% glucose and solidified with 0.8% plant agar. Material was grown under controlled light and temperature conditions: 24 °C, 16-h light/8-h dark photoperiod with a light intensity of 60 µmol photons m^−2^ s^−1^. *A. thaliana* plants (ecotype *Columbia*) were grown in controlled conditions of 8-h light/16-h dark with a light intensity of 100 µmol photons m^−2^ s^−1^ under stable temperature (23 °C in light/20 °C in dark).

### Gel electrophoresis

Total leaf extracts from transgenic *A. thaliana* plants were homogenized using plastic pestles in Laemmli buffer with 62.5 mM Tris pH 6.8, 10% glycerol, 5% SDS, 5% β-mercaptoethanol and loaded on a 15% (w/v) separating acrylamide gel (75:1 acrylamide/bis-acrylamide) with 6 M Urea. After SDS-PAGE gel electrophoresis, proteins were transferred by western blot on a polyvinylidene fluoride (PVDF) transfer membrane (Millipore) with the use of a Bio-rad blot system and developed using specific LHCSR and CP43 or CP47 antibodies produced in the laboratory.

### Thylakoid isolation and thylakoid fractionation

Thylakoids were purified from about 25 days old *A. thaliana* WT and transgenic plants (Berthold et al. 1981). Detached leaves from dark-adapted plants were harvested and homogenized in cold extraction buffer containing 0.02 M Tricine-KOH pH 7.8, 0.4 M NaCl, 0.002 M MgCl_2_, 0.5% milk powder, and protease inhibitors 5 mM ε-aminocaproic acid, 1 mM phenyl-methylsulfonyl fluoride and 1 mM benzamidine added right before the isolation. Homogenized leaves were then filtered, centrifuged at 1500×*g* for 15 min at 4 °C and then resuspended in a hypotonic buffer of 20 mM Tricine-KOH pH 7.8, 5 mM MgCl_2_, 150 mM NaCl and the pre-mentioned concentrations of protease inhibitors. Resuspended thylakoids were centrifuged for 10 min at 10,000×*g* (4 °C) followed by a second resuspension in a sorbitol buffer (10 mM HEPES–KOH pH 7.5, 0.4 M Sorbitol, 15 mM NaCl and 5 mM MgCl_2_). Thylakoid membranes were quantified and either used directly or stored in − 80 °C.

Solubilization was performed as in (Morosinotto et al. 2010; Pinnola et al. 2015a, b). Isolated thylakoids were resuspended in 20 mM HEPES–KOH, pH 7.5, 15 mM NaCl, 5 mM MgCl_2_ buffer at 1 mg Chl/mL and solubilized at 4 °C for 20 min in slow agitation with different amounts of α-DM ranging from 0.16 to 0.49% (w/v), always in the presence of 15 mM NaCl, 5 mM MgCl_2_ and 10 mM HEPES–KOH, pH 7.5. Unsolubilized thylakoids were pelleted by centrifugation at 3500×*g* for 5 min. Partially solubilized grana membranes were instead pelleted with a further 30 min centrifugation at 40,000×*g*. Solubilized complexes and small membrane patches remained in the supernatant. Membrane pellet was washed with 15 mM NaCl, 5 mM MgCl_2_ and 20 mM HEPES–KOH, pH 7.5, centrifuged for 30 min at 30,000×*g* and finally resuspended in 0.4 M Sorbitol, 10 mM HEPES–KOH, pH 7.5, 15 mM NaCl, 5 mM MgCl_2_ frozen in liquid nitrogen and stored at − 80 °C until use.

### Pigment-protein complexes separation with Deriphat-PAGE

Non-denaturating Deriphat-PAGE was performed as previously described (Peter et al. 1991) with some modifications: stacking gel of 3.5% (w/v) acrylamide (38:2 acrylamide/bis-acrylamide) and separating acrylamide gel was prepared at different fixed or gradient concentration depending on the purposes. Acrylamide concentrations are specified along the text. Thylakoids from wild type and transgenic plants corresponding to a final Chl concentration of 0.5 mg were washed with 5 mM EDTA and then resuspended in 10 mM HEPES pH 7.5. Samples were then solubilized with 0.8% n-Dodecyl α-d-maltoside and 10 mM HEPES pH 7.5 by vortexing thoroughly for 1 min. Solubilized samples were kept 10 min in ice and then centrifuged at 15,000×g for 10 min to pellet any insolubilized material and then loaded.

### Fluorescence measurements

In vivo Chl fluorescence was measured at room temperature after leaves were dark adapted for 45 min, by FC 800MF closed FluorCam Video-imaging system (Photon Systems Instruments, Czech Rep.) and PAM-100 (Walz, Germany) fluorometers. For every measurement a saturating pulse of 4000 µmol photons m^−2^ s^−1^ and actinic light with an intensity of 1200 µmol photons m^−2^ s^−1^ were applied. F_v_/F_m_ and NPQ parameters were calculated as (*F*_m_ − *F*_o_)/*F*_m_ and (*F*_m_–*F*_m_′)/*F*_m_′ respectively.

### Pigment composition analysis (HPLC)

*Arabidopsis thaliana* leaves or *P. patens* protonema tissue was frozen in liquid nitrogen and homogenized using plastic pestles. Pigments were extracted in 80% ice-cold acetone (buffered with NaHCO_3_) and analyzed by high-pressure liquid chromatography (HPLC) after a twostep centrifugation at 21,000×*g* for 10 min at 4 °C.

### 9-aminoacridin measurements

Intact chloroplasts were isolated from 4- to 5- week old *A. thaliana* (*npq4 *+ *LHCSR1*) plants or 6-days-old *P. patens psbs*-*lhcsr2 ko* (PzL_2_) based on the method from (Munekage et al. 2002). Tissue was homogenized using a potter in ice-cold buffer containing 330 mM sorbitol, 20 mM Tricine/NaOH (pH 7.6), 5 mM EDTA, 5 mM EGTA, 10 mM NaHCO_3_, 5 mM MgCl_2_, 0.1% (w/v) BSA and 1.87 mM Sodium l-ascorbate. The homogenized tissue was filtered through a nylon mesh and the filtrate was centrifuged at 2000×*g* for 5 min in a pre-chilled centrifuge (4 °C). The pellet was resuspended in an ice-cold buffer containing 300 mM Sorbitol, 10 mM HEPES/NaOH (pH 7.6), 5 mM MgCl_2_, 10 mM NaHCO_3_, 2.5 mM EDTA and 1.87 mM of Sodium l-ascorbate. Samples were kept on ice until right before the measurements. Chloroplasts were diluted to 25 µg/mL Chl in a buffer at room temperature containing 50 mM Tricine/NaOH (pH 8.0), 100 mM NaCl, 10 mM MgCl_2_ and 9-aminoacridine (2 µM) and measurements were performed at different light intensities 50, 200, 500 and 800 µmol photons m^−2^ s^−1^. Fluorescence measurements were recorded on a Fluoromax-3 (Horiba scientific), excitation wavelength 400 nm, emission measured at 430 nm. Electron transport was induced using the actinic light of a pulse-amplitude modulated fluorimeter (Heinz-Walz) equipped with a red filter (600–750 nm), 40 s of dark adaptation, 80 s illumination and 40 s of recovery in the dark.

## Results

### LHCSR1 expression in *A. thaliana npq4* mutant

The coding sequence of LHCSR1 was amplified from cDNA synthetized from *P. patens* protonema, cloned in the pH7WG2 vector under the control of the constitutive 35S promoter and used for *Agrobacterium*-mediated transformation of *npq4* mutant plants. The *npq4* mutants are devoid of qE due to the absence of PSBS. Transgenic seeds were collected and grown on hygromycin-B, resistant seedlings were transferred to soil, together with *A. thaliana* wild type (WT) and *npq4* control plants. Leaf extracts from *A. thaliana* genotypes and *P. patens* protonema tissue were analyzed by western blotting using α-LHCSR (Pinnola et al. 2013) and α-CP43 antibodies. WT and *npq4* plants showed no reaction with α-LHCSR while CP43 was detected in all samples (Fig. S1b). In *P. patens* both LHCSR1 and LHCSR2 were detected. In *A. thaliana* a single band, corresponding to LHCSR1, was obtained in hygromycin-resistant *A. thaliana* plants with a mobility matching the native LHCSR1 protein in *P. patens* thylakoid membranes. This suggests that *P. patens* LHCSR1 is both expressed and processed to its mature form in *A. thaliana*. The strongest LHCSR1 expressors among the complemented *A. thaliana npq4* lines were selected (C1, C3 and A5) and used to create homozygous lines, these lines contained multiple insertions which was established from the segregation pattern in later generations. It was verified that the T4 generation of line C1 was stable and further experiments, unless otherwise indicated, were performed on the T5 generation of this line. A quantitative western blot showed that these plants contain 82.8 ± 1.8% of LHCSR1 in comparison to the LHCSR1-only *P. patens psbs*-*lhcsr2 knock*-*out (ko)* (Fig. S2).

### LHCSR1 localization in *A. thaliana* thylakoid membranes

**Fig. 1:**
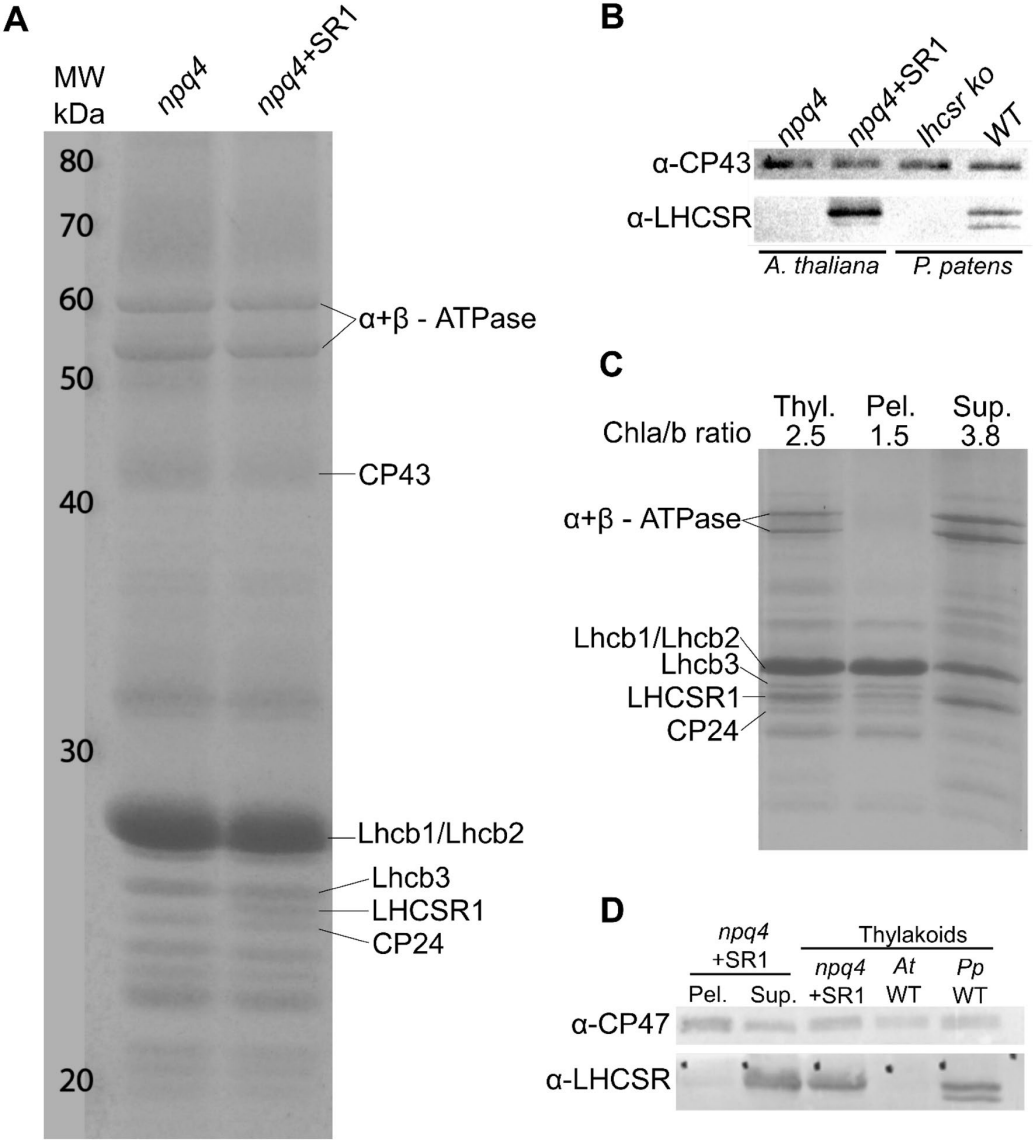
*A. thaliana* thylakoid membrane fractionation and analysis of LHCSR distribution in the individual fractions. **a** Coomassie-stained SDS-PAGE separation of thylakoid proteins isolated from *npq4* plants and *npq4* plants expressing LHCSR1 (*npq4 *+ SR1). LHCSR1 and other bands are indicated on the right side of the gel. **b** Western blot analysis of thylakoid proteins isolated from *A. thaliana npq4* plants and *npq4* plants expressing LHCSR. As control, thylakoids from *P. patens* WT and *lhcsr KO* were loaded. **c** Coomassie-stained SDS-PAGE of fractionated thylakoid membranes from *A. thaliana npq4 *+ LHCSR1 using 0.47% α-DM. Thylakoid membranes (Thyl.), pellet enriched in grana fractions (Pel.) and the supernatant enriched in stroma membranes (Sup.). Gels were loaded on Chl basis, 4 µg for thylakoids and 2.7 µg for both the pellet and supernatant fractions. The Chl a/b ratio is indicated above the gel. **d** western blot analysis of the fractionated thylakoid membranes and thylakoids from *A. thaliana npq4*, *npq4 *+ LHCSR1, WT and *P. patens* WT as controls

Thylakoid membranes from *A. thaliana npq4* plants expressing LHCSR1 and control *npq4* plants were purified and analyzed by SDS-PAGE (Fig. 1a). A band with the apparent molecular weight of LHCSR1 was present with a mobility between Lhcb3 and Lhcb6 (CP24) in the complemented plants but not in the background line *npq4*. The new band from the LHCSR1 expressing *A. thaliana* thylakoids was excised from gel and submitted to mass spectrometric analysis which yielded 10 peptides covering 58% of the mature protein sequence (Fig. S3). Each peptide matched the theoretical mass calculated from the DNA sequence, as predicted by ChloroP (Emanuelsson et al. 1999), implying no post-translational modifications were present within the identified fragments. No other identifiable changes in protein composition could be detected between the two genotypes (Fig. 1a). Furthermore, western blot analysis confirmed that the α-LHCSR antibody reacted against the LHCSR1 protein accumulated in the thylakoid membranes of the complemented plants (Fig. 1b) with the same electrophoretic mobility as the native protein from *P. patens.* LHCSR1 and LHCSR2 were detected in the WT *P. patens* thylakoids, but not in the *P. patens lhcsr1*-*lhcsr2 ko* thylakoid membranes (Fig. 1b).

The distribution of LHCSR1 in the thylakoid domains of *A. thaliana*, was assessed by thylakoid fractionation with n-dodecyl α-d-maltoside (α-DM) (Morosinotto et al. 2010; Pinnola et al. 2015a, b). The procedure yielded a pellet enriched in grana membranes and a supernatant comprising the stroma lamellae. The *Coomassie*-stained SDS-PAGE gel confirmed PSI and ATPase were in the stroma-derived supernatant fraction while the PSII core subunit as well as LHCII and Lhcb6 (CP24) were enriched in the pellet, i.e. the grana partitions (Fig. 1c). A band with the apparent molecular weight corresponding to LHCSR1 was highly enriched in the supernatant, suggesting that recombinant LHCSR1 was localized in thylakoid stroma-exposed membranes of complemented *A. thaliana npq4*. This was confirmed by western blot (Fig. 1d) and step-solubilization with increasing concentrations of α-DM detergent, namely 0.16, 0.24, 0.32, 0.39 and 0.47% α-DM (Fig. S4). Immunoblotting showed that LHCSR1 was already enriched in the stromal fraction at 0.16% α-DM with a Chl *a*/*b* ratio > 6.0 and a polypeptide composition, including PSI and ATPase, typical of stroma membranes (Fig. S4a, b). Low amounts of LHCSR1 were found in the pellet fractions up to 0.32% α-DM suggesting that the protein might also be localized in the margins of *A. thaliana* thylakoids.

### Pigment binding to recombinant LHCSR1 and their spectra

**Fig. 2:**
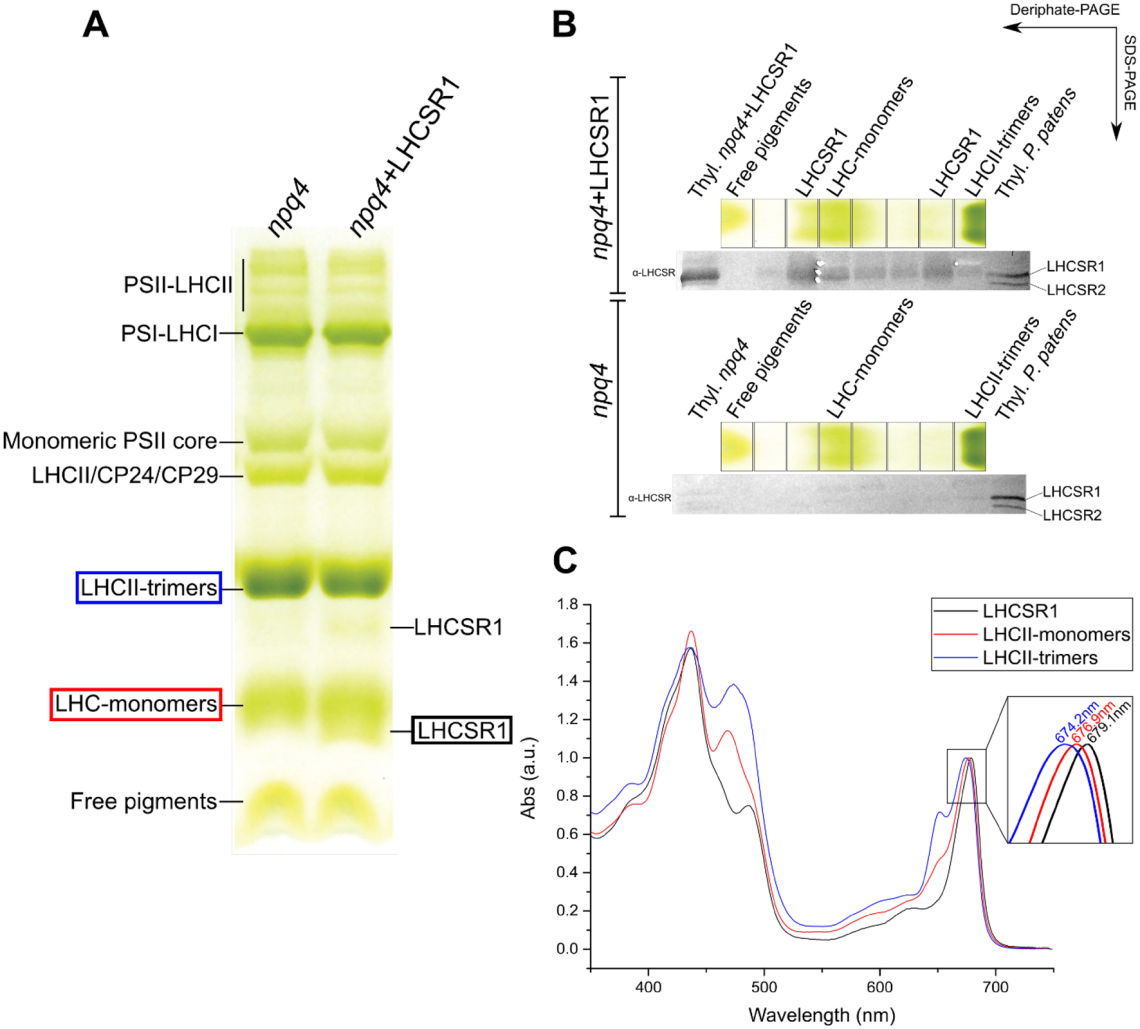
Deriphat-PAGE analysis of *A. thaliana* thylakoid membrane protein complexes. **a** Thylakoid membranes (30 μg of Chl) of *npq4* and *npq4 *+ LHCSR1 plants solubilized with 0.8% (w/v) α-DM were subjected to Deriphat-PAGE. PSII and PSI complexes, together with various LHCs are indicated on the left side of the gel. Complexes more abundant in *npq4 *+ LHCSR1 than in *npq4* plants are labeled as LHCSR1 on the right side of the gel. Colored rectangles correspond to the bands used for the absorption spectra analysis in (panel **c**). **b** Deriphat-PAGE (7%) of unstacked thylakoids from *A. thaliana* WT and *npq4 *+ LHCSR1, solubilized in 0.8% α-DM. Bands were eluted in 10 mM HEPES/0.03% α-DM. Eluted fractions were loaded on SDS-PAGE and immunoblotted against α-LHCSR. **c** Absorption spectra of the bands taken from the Deriphat-PAGE (panel **a**), LHCSR1, LHCII-monomers and LHCII-trimers of *A. thaliana npq4 *+ LHCSR1

LHCSR is a pigment-binding protein in *C. reinhardtii* and *P. patens* (Bonente et al. 2011; Pinnola et al. 2013). To verify that recombinant LHCSR1 expressed in *A. thaliana* did actually refold properly with pigments, thylakoid membranes were analyzed by Deriphat PAGE (Fig. 2a). Although the protein composition was similar between the two genotypes, *A. thaliana npq4 *+ LHCSR1 did contain two additional bands with respect to *A. thaliana npq4*, migrating, respectively, just below the monomeric LHC band and in between LHC monomers and trimers. Gel slices were excised from the gel and further separated by denaturing SDS-PAGE, followed by western blotting. The two “additional” bands in the gel from *A. thaliana npq4 *+ LHCSR1 showed strong reaction towards the α-LHCSR antibody (Fig. 2b). Fainter reactions were also obtained with fractions from in between the two bands but not with those at lower or higher mobility, suggesting that LHCSR1 migrated initially as a dimeric Chl binding protein which partially dissociated into monomers during solubilization and/or electrophoretic migration.

LHCSR proteins have a characteristically red-shifted absorption spectrum with respect to other LHC proteins (Bonente et al. 2011; Pinnola et al. 2013, 2015b). Absorption spectra recorded from extracted gel bands showed a red-shifted *Q*_y_ peak at 679.1 nm with respect to LHCII trimers (674.2 nm) and LHC monomers (676.9 nm), typical for LHCSR1 (Bonente et al. 2011; Pinnola et al. 2017) (Fig. 2c). Also, the LHCSR1-containing band was depleted of Chl *b* with respect to the bands from other LHCs, thus copying the properties of recombinant LHCSR proteins either refolded in vitro or expressed in tobacco (Bonente et al. 2011; Pinnola et al. 2015b).

### NPQ activity of LHCSR1 in *A. thaliana*

**Fig. 3:**
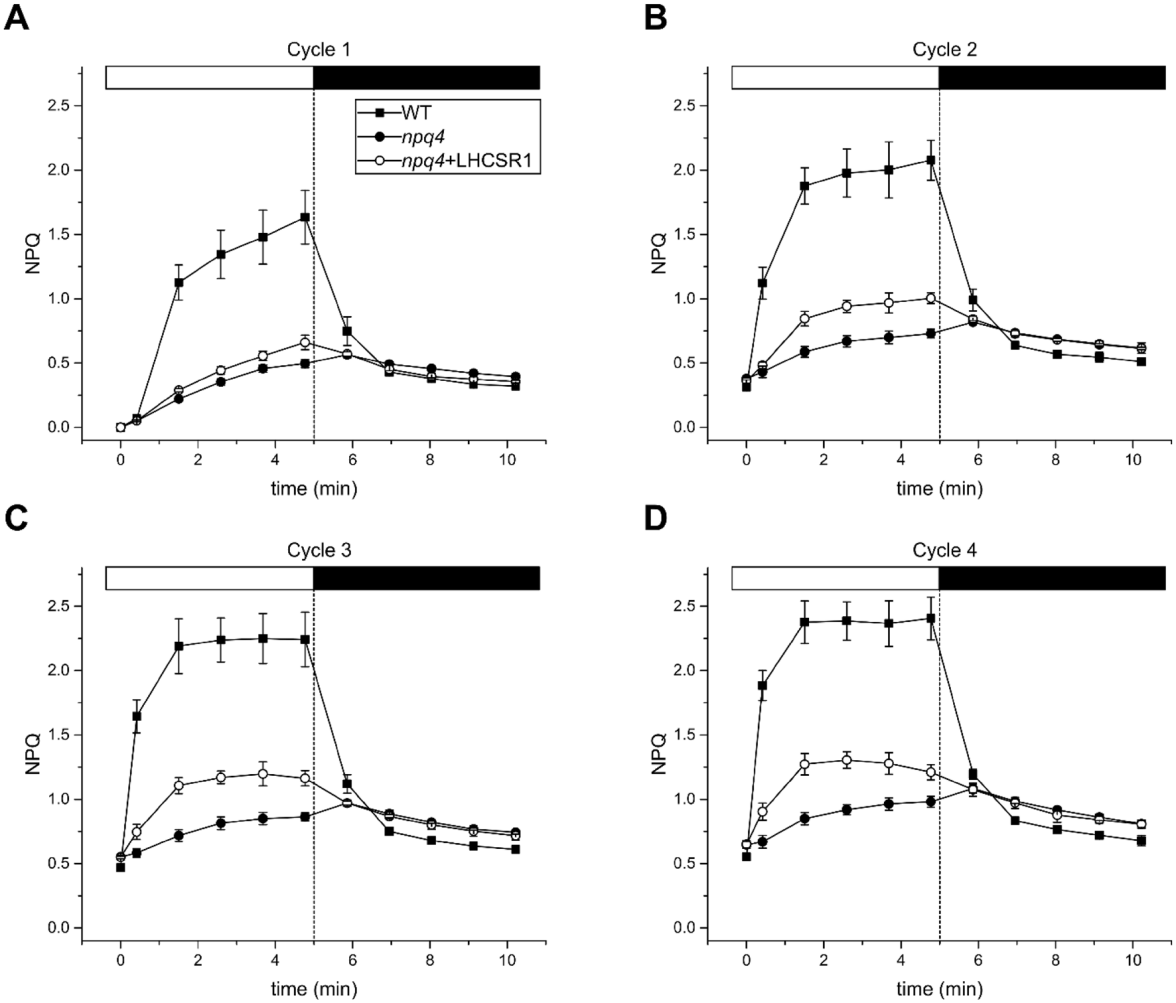
NPQ kinetics (*n *= 4) in *A. thaliana* WT, *npq4* and *npq4 *+ LHCSR1. WT (Black squares), *npq4* (black circles) and *npq4 *+ LHCSR1 (open circles). Plants were dark adapted for 45 min before the measurement, 4 cycles of 5 min actinic light (1200 µmol photons m^−2^ s^−1^) and 5 min dark, as described in the M&M. The four cycles are depicted by **a**–**d**, respectively

As previously mentioned, the NPQ activity of PSBS and LHCSR is additive and independent in *P. patens* plants (Alboresi et al. 2010; Gerotto et al. 2012). It was tested whether LHCSR1 could confer a light dependent in vivo quenching activity in the *A. thaliana npq4* mutant. Therefore, the Chl fluorescence quenching of *A. thaliana* WT, *npq4* plants and the complemented lines were measured using Chl fluorescence imaging. The protocol consisted of a 45 min dark adaptation of the leaves, followed by 5 min white actinic light (1200 µmol photons m^−2^ s^−1^) and 5 min of dark recovery (Fig. 3). When the protocol was applied to dark-adapted leaves, only a small difference in quenching activity was observed, suggesting the expression of LHCSR1 did not confer significant NPQ activity (Fig. 3a). However, when the same protocol was applied for the second-time, larger differences between *npq4* and *npq4 *+ LHCSR1 were observed (Fig. 3b). Two additional cycles of NPQ induction and relaxation were applied, where the differences were even further pronounced between the second and third measurement, the NPQ was very similar between the third and the fourth measurement (Fig. 3c, d). Interesting to note is that the *npq4* mutant showed a characteristic transient increase of quenching at the first point in the dark after switching off the actinic light, this jump was not detected in plants containing PSBS nor was it detected in lines expressing LHCSR1 (Fig. 3a–d). Since the accuracy of these NPQ measurements depends on that of the Fm measurements. All the Fv/Fm values of the different NPQ measurements were included in Tables S1 and S2. *F*_v_/*F*_m_ values were found to be very similar for all the different LHCSR1 expressing lines (i.e. below 2%).

### LHCSR1 and zeaxanthin synthesis

Since Zea has a major influence on the quenching activity of LHCSR1 (Pinnola et al. 2013), the slow onset of LHCSR1-dependent NPQ activity in *A. thaliana* suggests that Zea accumulation might be limiting. Therefore, the leaf pigment content was determined by HPLC analysis of the *npq4* and two independent *npq4 *+ LHCSR1 lines during two cycles of 10 min illumination followed by a 10 min dark relaxation. Zea accumulated to the same level in both genotypes (Table S3) at the end of each dark or light phase while the 10-min dark periods did not allow for a decrease in Zea level. We conclude that the repeated cycles of illumination (Fig. 3) lead to an increased NPQ activity, which is consistent with the accumulation of Zea (Table S3).

### Correlation between LHCSR1 accumulation level and NPQ activity

**Fig. 4:**
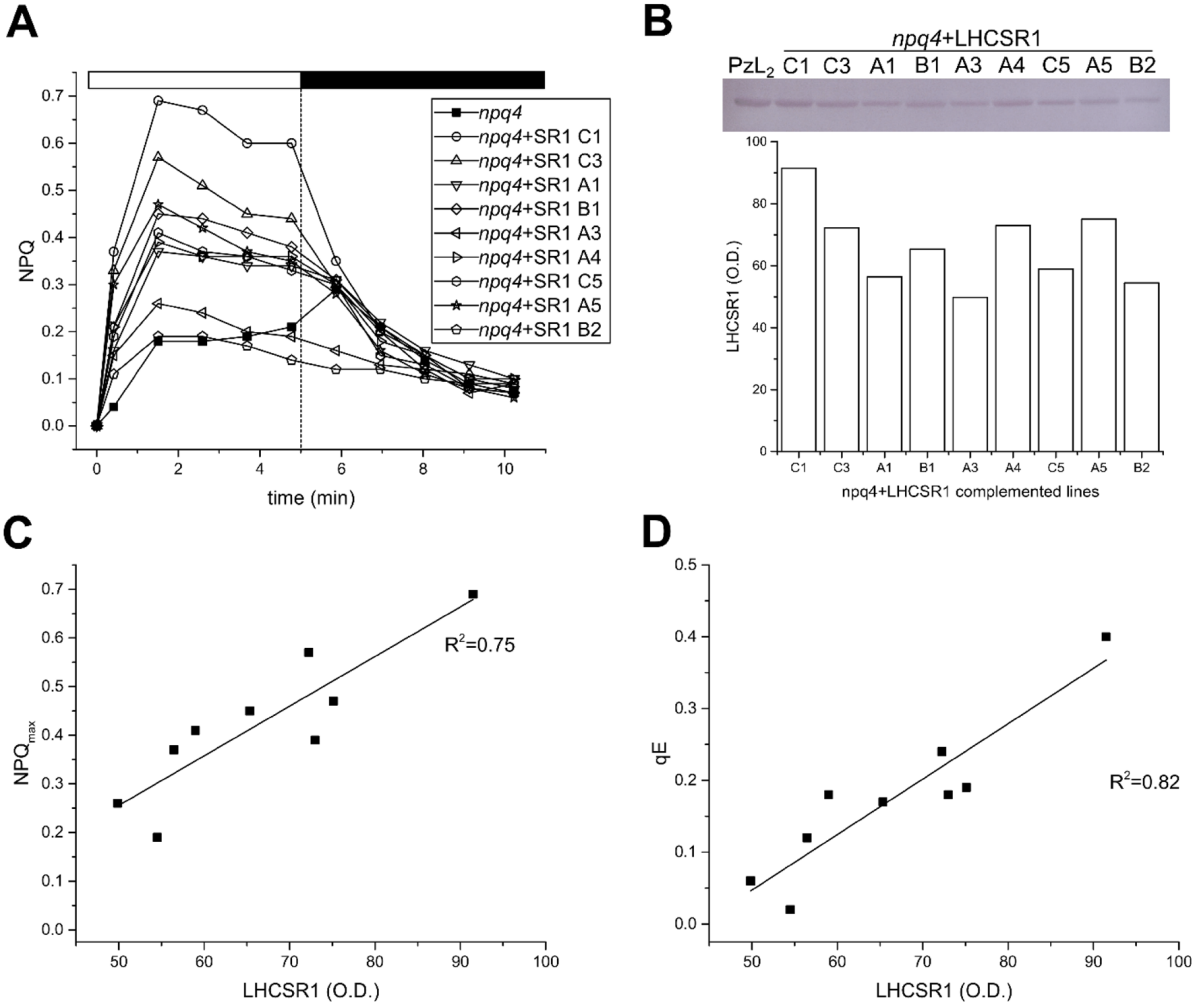
Correlation between NPQ activity and LHCSR1 accumulation. NPQ measurements of the T2 generation of the *npq4 *+ LHCSR1 lines (*n *= 9). **a**. Leaves were dark adapted for 45 min, pre-treated with 1200 µmol photons m^−2^ s^−1^ of actinic light for 15 min and left to relax in the dark for 10 min before the NPQ measurement **b** After the NPQ measurement total leaf extracts from each line were loaded on an SDS-PAGE on a basis of 0.75 µg Chl and immunο-blotted against α-LHCSR antibodies. Thylakoids from *P. patens psbs*-*lhcsr2 ko* (PzL_2_) were loaded as a control. The O.D. of LHCSR1 was determined. **c** Protein level plotted with the maximum NPQ, yielding a positive correlation of *R*^2^ = 0.75. **d** Correlation between qE and the protein level (*R*^2^ = 0.82). qE recovery is calculated as the NPQ of the last point in the light phase minus the second point in the dark phase

**Fig. 5:**
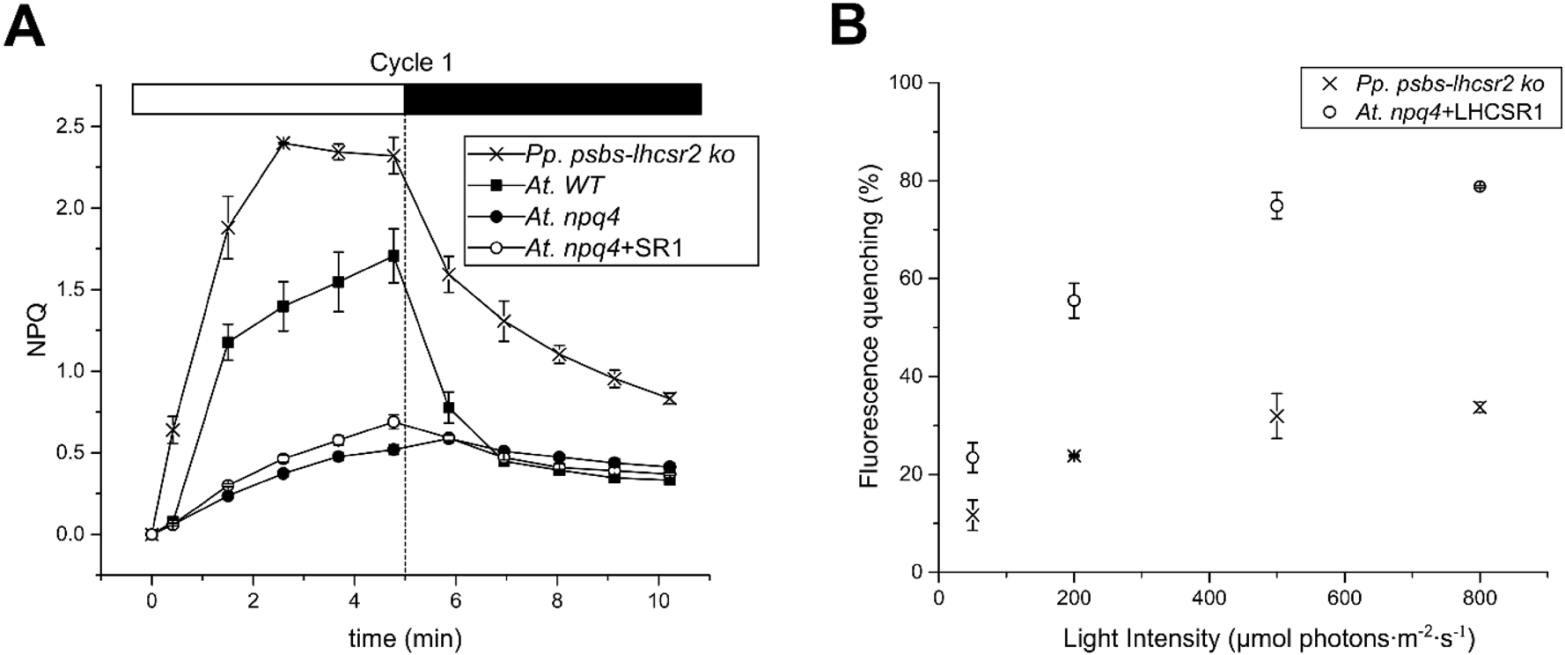
Comparison of ΔpH and NPQ between *P. patens psbs*-*lhcsr2 ko* and *A. thaliana.***a** Comparison of NPQ (*n *= 4) between *A. thaliana* WT, *npq4*, *npq4 *+ LHCSR1 (*npq4 *+ SR1) and *P. patens psbs*-*lhcsr2 ko*. Fourth cycle of NPQ measurements at 1200 µmol photons m^−2^ s^−1^ (5 min light and 5 min dark relaxation). **b** 9-aminoacridin measurements (*n *= 3) in isolated chloroplasts of *A. thaliana npq4 *+ LHCSR1 and *P. patens psbs*-*lhcsr2 ko* at different light intensities (50, 200, 500 and 800 µmol photons m^−2^ s^−1^ of red light)

To verify whether NPQ activity correlated with the amount of LHCSR1, nine lines with different levels of NPQ were selected (Fig. 4a). Total leaf extracts were titrated with an α-LHCSR polyclonal antibody (Fig. 4b). Both qE and total NPQ activities linearly correlated with the level of LHCSR1. An estimation of the qE was determined by differences between NPQ values recorded at the end of the 5-min light period and upon 2 min of dark relaxation, allowing for a rapid estimation of qE activity (Dall’Osto et al. 2014). The NPQ activity per LHCSR1 unit was lower in *A. thaliana* with respect to *P. patens* since LHCSR1-only mosses did show an NPQ score threefold higher than the complemented *A. thaliana npq4* lines (Fig. 5a), while the level of LHCSR1 in *P. patens psbs*-*lhcsr2 ko* was only 1.2-fold higher (Fig. S2).

### LHCSR1-dependent NPQ in *A. thaliana*: dependence on light intensity

**Fig. 6:**
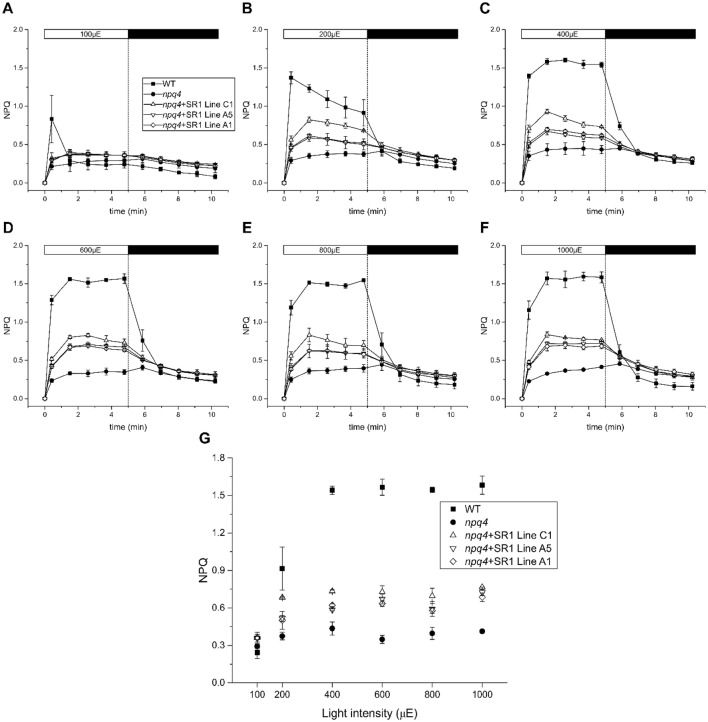
NPQ activity of *npq4 *+ LHCSR1 lines in various light intensities. Three different *A. thaliana npq4 *+ LHCSR1 lines with high and intermediate NPQ activation (line C1, A1 and A5) were tested in a variety of actinic light intensities. Leaves (*n *= 3) were dark adapted for 45 min, pre-treated with 800 µmol photons m^−2^ s^−1^ of actinic light for 15 min and left to relax in the dark for 10 min before the NPQ measurement. Each measurement corresponds to one single NPQ cycle of 5 min different with different actinic light intensities and 5 min dark recovery. The actinic light intensities used were: 100, 200, 400, 600, 800 and 1000 µmol photons m^−2^ s^−1^ (µE) from **a**–**f**, respectively. Leaves from *A. thaliana* WT and *npq4* were used as controls. **g** NPQ of the last point in the light plotted against the different light intensities

In order to investigate the reasons for the low LHCSR1 activity in *A. thaliana* versus *P. patens*, we verified the hypothesis that mosses might differ from *A. thaliana* for their light intensity dependence of LHCSR1 activity consistent with moss adaptation to shaded habitats. To this aim, three *npq4 *+ LHCSR1 lines with high and intermediate NPQ activity at 800 µmol photons m^−2^ s^−1^ were selected and measured at a series of actinic light intensities: low light intensity (100 µmol photons m^−2^ s^−1^) up to 1000 µmol photons m^−2^ s^−1^. Before each measurement, leaves were dark-adapted for 45 min, pre-treated with actinic light (800 µmol photons m^−2^ s^−1^) for 15 min in order to accumulate equal Zea levels and left to relax for 10 min in the dark. Leaves from *A. thaliana* WT and *npq4* plants of the same age were used as controls. At the lowest light intensity, transient NPQ was observed in all *A. thaliana* genotypes, which rapidly dropped, likely due to activation of the ATPase dissipating the ΔpH for ATP synthesis (Fig. 6). However, as the intensity of actinic light increased, plants activated NPQ and the LHCSR1 complemented lines already showed activity at 200 µmol photons m^−2^ s^−1^. Peak activity was reached after 2–3 min light exposure and the NPQ level was maximal at 400 and 600 µmol photons m^−2^ s^−1^, with lower values at both lower and higher actinic light intensities. WT *A. thaliana*, besides showing at least twofold higher NPQ values, also did show strikingly different NPQ kinetics, monotonously rising under actinic light conditions and only relaxing when light was switched off. The partial relaxation of NPQ under actinic light could be explained by a relaxation of lumen acidity after 3 min of light treatment.

To verify whether the activation of LHCSR1 might be affected by the amplitude of the pH gradient formation, we proceeded to measure the ΔpH through thylakoid membranes at different light intensities. To this aim, chloroplasts of *A. thaliana npq4 *+ LHCSR1 plants and *P. patens psbs*-*lhcsr2 ko* were illuminated in the presence of the fluorophore 9-aminoacridine (9-AA). 9-AA fluorescence is quenched upon protonation when the chemical diffuses through the thylakoids into the lumen dependent on the trans-membrane pH gradient. Figure 5b shows the 9-AA fluorescence quenching in *A. thaliana* versus *P. patens* at different light intensities, implying a different capacity of building up a trans-thylakoid pH gradient between the two organisms. Despite the fact that *A. thaliana* was able to reach higher ΔpH levels than *P. patens,* the NPQ activity of LHCSR1 in *A. thaliana* was lower with respect to *P. patens* (Fig. 5a).

### LHCSR1 expression and NPQ activity in xanthophyll biosynthesis mutants

The above results imply that LHCSR1 proteins expressed in *A. thaliana npq4* can partially complement the lack of PSBS. When purified from *Physcomitrella patens*, LHCSR1 binds lutein (Lut) and Vio, part of which are substituted by Zea upon high light treatment (Pinnola et al. 2013). To identify the role of these xanthophylls for activation of LHCSR1 in *A. thaliana* we proceeded with the transformation of the *lhcsr1*-gene in the following *A. thaliana* double mutants: (*i*) *npq1npq4*, unable to accumulate Zea due to the lack of violaxanthin de-epoxidase (*vde*); (ii) *npq2npq4*, a mutant accumulating Zea due to the absence of zeaxanthin epoxidase (*zep*) (*iii*) *lut2npq4*, the lutein-less genotype defective in the lycopene ε-cyclase activity, which compensates missing Lut with increased levels of Vio.

**Fig. 7:**
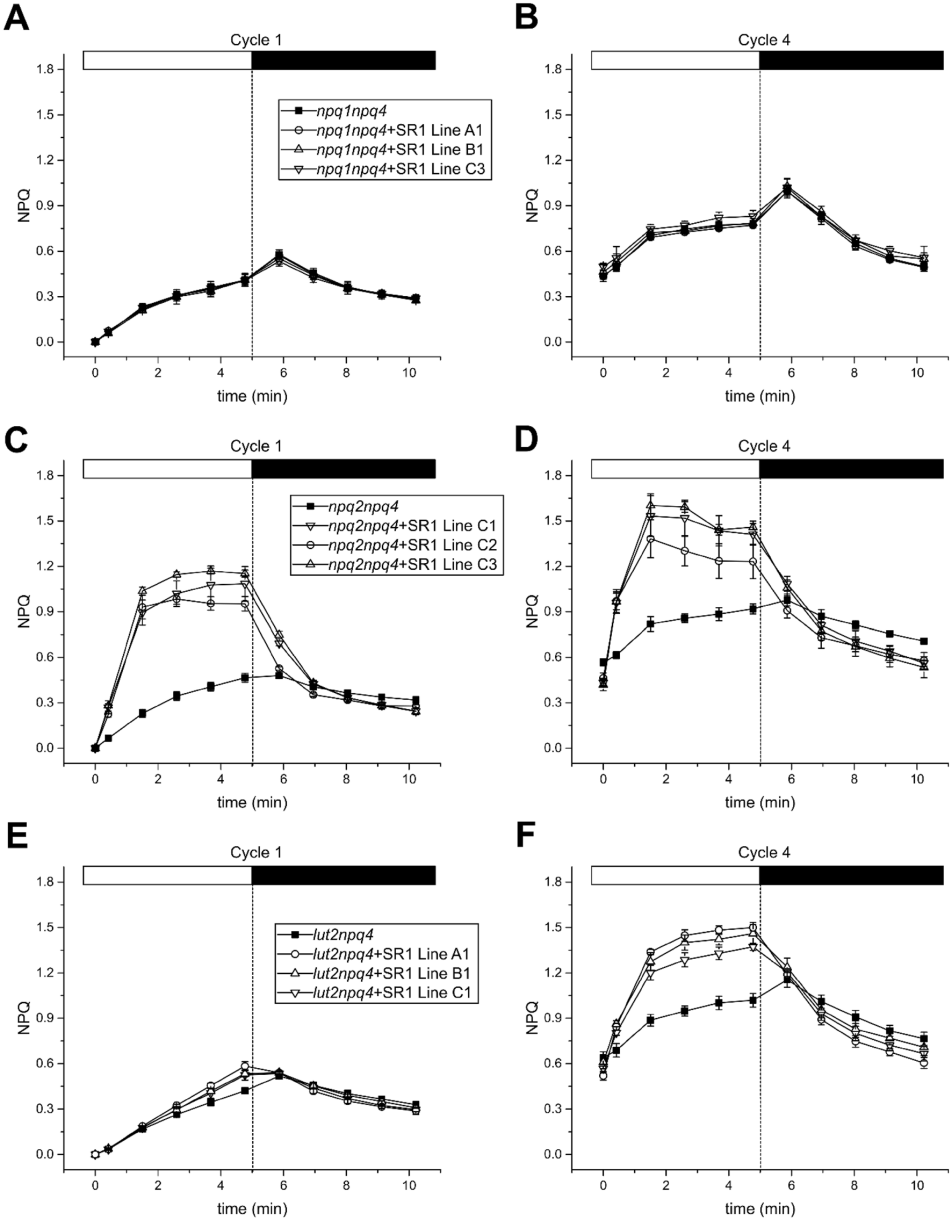
NPQ measurements in *A. thaliana* mutants, lacking specific xanthophyll’s, complemented with LHCSR1. Four successive NPQ cycles were measured (*n *= 3) as described in M&M. The first and fourth cycle are shown for each complemented mutant. **a**, **b** first and fourth NPQ measurement in *npq1np4* and three independent *npq1npq4* lines complemented with LHCSR1 (*npq1npq4 *+ SR1). **c**, **d** first and fourth NPQ measurement in *npq2npq4* and three *npq2npq4* lines complemented with LHCSR1 (*npq2npq4 *+ SR1). **e**, **f** first and fourth NPQ measurement in *lut2npq4* and three *lut2npq4* lines complemented with LHCSR1 (*lut2npq4 *+ SR1)

Transformation of the *npq1npq4* mutant with LHCSR1 and selection in hygromycin yielded 16 stable lines, which accumulated LHCSR1 as assessed by western blot analysis (Fig. S5b), implying LHCSR1 can be expressed and accumulated in the absence of Zea (Fig. S5b). No major differences in the size or shape of the transformed plants were detected (Fig. S5a). The NPQ activity of *npq1npq4* plants and three independent complemented lines was measured by video-imaging following the initial protocol (see M&M). The quenching activity of the complemented lines was the same as in the *npq1npq4* background and did not increase during the subsequent cycles of illumination, failing to reveal any difference between *npq1npq4* (i.e. control) and the complemented *npq1npq4 *+ LHCSR1 plants (Fig. 7a, b; Fig. S6a–d). This result is in agreement with previous reports in the homologous system *P. patens* showing that LHCSR1 requires Zea for quenching (Pinnola et al. 2013).

The *npq2* mutant lacks Vio and accumulates full levels of Zea as well as Lut (Niyogi et al. 1998; Peers et al. 2009). Complementation of the *npq2npq4* mutant yielded three plants accumulating LHCSR1 (Fig. S7). NPQ activity differed with respect to *npq4 *+ LHCSR1 in that it appeared already during the first cycle of illumination in dark-adapted plants (Fig. 7c, d; Fig. S8a–d). Furthermore, the total amount of NPQ in these plants was much higher, reaching up to 70% of *A. thaliana* WT. These observations indicate that the build-up and level of Zea are one of the limiting factors in the NPQ activity of LHCSR1. During the fourth cycle the maximal NPQ was reached after 2 min and showed the same partial relaxation kinetics as the *npq4 *+ LHCSR1. This relaxation, however, could already be observed during the third cycle in the *npq2npq4* complemented lines, but not in *npq4 *+ LHCSR1.

LHCSR1 expression in the *lut2npq4* yielded no major phenotypic differences between control and LHCSR1–complemented *lut2npq4* plants (Fig. S9a). Total leaf extracts from the complemented lines were analyzed by western blot with the α-LHCSR antibodies (Fig. S9b), showing that the protein was processed to its mature form without Lut. Using the 4-cycle actinic light protocol, the control and complemented lines were essentially indistinguishable in the first cycle (Fig. 7e). However, upon the second cycle the curves of the different genotypes became more shifted towards higher values with the exception of *lut2npq4*, which remained unchanged (Fig. S10). Two additional cycles further increased the difference in NPQ between *lut2npq4* and the complemented lines (Fig. 7f). Two features characterized these measurements with respect to the *npq4 *+ LHCSR1 genotype: first, the higher level of qE obtained in the *lut2npq4 *+ LHCSR1 with respect to *npq4 *+ LHCSR1; second, that the maximal NPQ values were obtained at the end of the illumination (5 min) rather than at the second minute as previously observed with the *npq4 *+ LHCSR1 and *npq2npq4 *+ LHCSR1 genotypes, consistent with a delay in reaching full de-epoxidation of Vio bound to slowly exchanging binding sites (Morosinotto et al. 2002).

## Discussion

While expression in tobacco has proven instrumental for purification of the LHCSR1 protein for biochemical, spectroscopic and structural studies (Pinnola et al. 2015b, 2016, 2017; Kondo et al. 2017, 2019), *A. thaliana* is a choice for functional studies due to the availability of a large collection of mutants and the easiness of transformation procedures. Here, we show that the moss LHCSR1 protein can be expressed in *A. thaliana* to form a pigment-protein complex indistinguishable from the holoprotein purified from *P. patens* as judged from the mass spectrometric analysis and visible absorption spectra (Fig. 2c, S3). Also consistent with previous reports, LHCSR1 was found both as a monomer and dimer in non-denaturing green gels, suggesting its aggregation state in thylakoids is a dimer which, in part, monomerizes during solubilization and fractionation (Pinnola et al. 2015b). LHCSR1 purified from *P. patens*, binds Chl *a*, Lut and Vio with sub-stoichiometric levels of Chl *b,* where Vio is largely substituted by Zea during high light treatment (Pinnola et al. 2013, 2015b). Here, we show that LHCSR1 from *P. patens* can be expressed in *A. thaliana npq4* mutants at levels comparable to that found in moss. Due to the absence of PSBS, the host lines did not show NPQ activity upon high light treatment (Li et al. 2000). Thus NPQ detected in complemented plants can be attributed to LHCSR1 based on the following observations: (i) quenching was only observed in LHCSR-complemented genotypes (Fig. 3); (ii) quenching was proportional to the level of LHCSR1 accumulation (Fig. 4); (iii) no LHCSR1-dependent quenching activity was observed in the *npq1npq4* background, lacking Zea (Fig. 7a, b), in agreement with the observation that the *vde* ko mutant in *P. patens* lost 95% of quenching activity (Pinnola et al. 2013); (iv) higher and fast-developing quenching was observed upon expression in the *npq2npq4* background lacking Vio and constitutively accumulating Zea (Fig. 7c, d). All these features closely reproduce the properties of LHCSR1 activity in the moss, implying the observed NPQ could be *bona fide* attributed to LHCSR1. LHCSR1 was correctly addressed to the thylakoid membranes with an apparent molecular weight identical to the LHCSR1 of *P. patens,* as observed in SDS-PAGE gels (Fig. 1), implying a correct targeting and processing of the pre-protein encoded by the construct. We then proceeded to identify the factors which determine the level of NPQ activity, including the accumulation in the thylakoids, the availability of the Zea co-factor and the co-localization with PSII whose fluorescence is quenched during NPQ. The level of LHCSR1 in *A. thaliana npq4* was slightly lower (82.8 ± 1.8%) with respect to the *P. patens psbs*-*lhcsr2 ko*. Yet, while LHCSR1 is the major contributor to NPQ activity in moss (Alboresi et al. 2010) providing an NPQ activity of 3.2, the NPQ activity in *A. thaliana* was lower. Besides the lower levels of Zea found in *A. thaliana*, this can be explained by (i) a lower level of LHCSR1 activation by a difference in lumen acidification; (ii) a different localization in thylakoids with respect to the PSII antenna system, which is the major fluorescence emitter in vivo; or (iii) the lack of one or more interaction partner(s) acting as a docking site for connecting the quenching site within the LHCSR1 to the PSII antenna system. To test the first hypothesis, we proceeded to determine the ΔpH in *npq4 *+ LHCSR1 and *P. patens psbs*-*lhcsr2 ko* chloroplasts by the 9-AA quenching method and showed that ΔpH formation is higher in *A. thaliana* with respect to *P. patens*. This would suggest that the lower NPQ in *A. thaliana* might be due to over-acidification of the lumen. LHCSR1, however, was not active at 100 µmol photons m^−2^ s^−1^ in *A. thaliana*, a light intensity where the ΔpH is comparable to that found in *P. patens.* The highest quenching activity of LHCSR1 in *A. thaliana* was found at 400 and 600 µmol photons m^−2^ s^−1^, light intensities where the ΔpH is already much higher in *A. thaliana* in comparison to *P. patens.* Making it unlikely that over-acidification is the reason for the lower quenching activity. Hypothesis (ii) appears to be relevant in determining a reduced NPQ since the membrane fractionation experiment located LHCSR1 in the stroma membranes (Fig. 1c, d), consistent with previous findings in the moss. It should be noted that a large fraction of LHCII is located in moss stroma membranes (Pinnola et al. 2015b), while higher plants show extreme lateral heterogeneity with LHCII being located almost exclusively in the grana (Bassi et al. 1988; Pribil et al. 2014). Thus, interaction between LHCSR1 and PSII antennas appears to be restricted to grana margins, implying that only a low fraction of PSII supercomplexes might be involved. This is likely to decrease the quenching efficiency of *npq4 *+ LHCSR1 plants with respect to WT *A. thaliana*, with PSBS localized in grana partitions together with PSII antenna (Pinnola et al. 2015b). Hypothesis (iii) is synergic with (ii). In fact, work in *A. thaliana* (Pietrzykowska et al. 2014) and *C. reinhardtii* (Elrad et al. 2002; Ferrante et al. 2012) has shown that quenching requires specific members of the LHC protein family with which PSBS and LHCSR interact (Girolomoni et al. 2016). It is well possible that one or more LHCSR1-interacting proteins in moss are not conserved in *A. thaliana* and/or that the interaction is partially impaired.

In addition to differences in amplitude, the kinetics of LHCSR1-dependent quenching is also different in transgenic *A. thaliana* versus *P. patens* in some aspects: first, quenching is activated only upon pre-treatment with high light while in the moss it is evident at the first light exposure of dark-adapted mosses (Fig. S11). Explanation of this behavior is provided by the results of complemented *npq1npq4* and *npq2npq4* genotypes: plants from the former genotype showed no quenching activity due to the absence of Zea, despite LHCSR1 accumulation in the thylakoids. Plants from the latter genotype, the *npq2npq4* which are fully endowed with Zea, not only show a higher quenching activity, but also, a faster activation, i.e. at the first cycle of illumination (Fig. 7c) rather than at the third as in the *npq4* plants expressing LHCSR1. Even though the *npq2npq4* mutant contains 2.5 folds more Zea in comparison to high light adapted *P. patens* (Dall’Osto et al. 2005; Pinnola et al. 2013), the level of NPQ was lower than found in both *P. patens* LHCSR1-only or *A. thaliana* WT, implying that Zea was not the only limiting factor for the quenching activity of LHCSR1 in *A. thaliana*. Interestingly, the kinetics of the De-epoxidation Index (DEP) are very similar to the kinetics of quenching in both *A. thaliana* and *P. patens*: in *A. thaliana* the maximal DEP was reached after 10 min (Dall’Osto et al. 2017), in *P. patens* a DEP comparable to the maximum of *A. thaliana* was already reached after 1.5 min (Fig. S12). This suggests that there is simply not enough Zea in *A. thaliana* to completely activate LHCSR1, which is consistent with the enhanced activity of LHCSR1 in the *npq2npq4* background where Zea availability was constitutive rather than induced by light exposure. The *lut2npq4* complemented lines, lacking Lut, showed activity in NPQ, meaning that Lut is not an absolute requirement for the in vivo quenching in LHCSR1. This is consistent with the recent finding that the major quenching mechanism in isolated LHCSR1 is energy transfer from Chl to S1 state of Zea followed by rapid relaxation to ground state, while transient formation of Lut radical cation was low (Pinnola et al. 2016). In the *lut2npq4* complemented lines the peak NPQ activity was observed at later times upon each onset of the actinic light. While the NPQ peak was observed after 2 min of light exposure in *npq4 *+ LHCSR1, the NPQ of *lut2npq4 *+ LHCSR1 steadily raised till the end of the light phase. This behavior can be explained based on the enhanced Vio content in the L2 binding site, due to compensation for the missing Lut (Pogson et al. 1996; Dall’Osto et al. 2006). Vio is an inhibitor of quenching reactions (Niyogi et al. 1998; Ruban et al. 1998) and replaces Lut in sites L1 and L2 of LHCII proteins (Croce et al. 1999; Dall’Osto et al. 2006). The kinetic difference can be explained with two independent events of xanthophyll exchange: one at site L1, which substitutes Vio for Zea, while the second event is the exchange at site L2 which, are kinetically different in LHC proteins (Morosinotto et al. 2002). Since occupation of L1 site by Lut or Zea is essential for the NPQ activity (Dall’Osto et al. 2006), the onset of NPQ was slower in *lut2npq4 *+ LHCSR1 with respect to *npq2npq4 *+ LHCSR1 or *npq4 *+ LHCSR1 which have Lut in site L1 already in the dark and only need to perform the Vio to Zea exchange in site L2. Future research will need to devise new methods for assessing the xanthophyll composition of LHCSR1 in real time as well as other LHC proteins essential for quenching reactions in plants, mosses and algae since the exchange might be fast and reversible in minutes. It remains to be explained why the *npq2npq4 *+ LHCSR1 or *npq4 *+ LHCSR1 show an NPQ kinetic rapidly climbing to a peak and then relaxing or remaining constant during the remaining light period. We suggest this depends on the ΔpH +Δψ gradient through the thylakoid membrane that appears to be different in *A. thaliana* versus *P. patens*. 9AA quenching showed a lower ΔpH contribution in *P. patens* and yet LHCSR1 might respond to Δψ as well. Upon transition from dark to excess light, a transient lumen acidification is reached due to the contribution of cyclic electron flow, recycling excess reducing power into over-reduction of plastoquinol and additional proton transport (Munekage et al. 2004). The ΔpH and/or Δψ past transient cyclic burst might be insufficient to sustain full LHCSR1 activation in *A. thaliana*.

## Conclusion

We show that heterologous expression of LHCSR1 in *A. thaliana npq4* mutant yields a pigment-binding protein with properties reproducing those of LHCSR1 from the homologous system *P. patens*. The protein is active in NPQ, yet the induction requires sustained light treatment due to the need for Zea build-up. Reasons for a decreased NPQ include (a) insufficient Zea accumulation in *A. thaliana* with respect to *P. patens* for full NPQ activity and (b) the localization of the protein in the stromal membranes of thylakoids which is rich in highly fluorescent LHCII in mosses but not in plants (Pinnola et al. 2015a). The level of quenching in *npq2npq4 *+ LHCSR1 (endowed with full Lut and Zea levels) recovered up to 70% with respect to *A. thaliana* WT, proving that LHCSR1 can be highly functional in vascular plants. Furthermore, we prove that this system is sensitive to physiological differences which makes *A. thaliana* an excellent organism for the analysis of LHCSR activity. Indeed, we could assess that Lut was not an absolute requirement for in vivo quenching in LHCSR1, since quenching activity was obtained in *lut2npq4 *+ LHCSR1 plants.

The primary target of plants is survival; thus, they favor thermal dissipation over fast growth. But an NPQ mechanism with low activity in moderate light intensity and full activation in extreme stress conditions only, might allow for optimizing both growth and stress resistance. This could be the case for the LHCSR1 protein expressed in the heterologous systems which exhibits an activity, even if low in the WT and higher in the *npq2* background, in stressing conditions. Future work will evaluate the growth performance (productivity) of these plants in different constant light condition as well as in fluctuating light which is the most stressing condition and mimics the natural environment.

Finally, since the NPQ activity of LHCSR1 and PSBS is cumulative, suggesting they have different interaction partners, future work with deletion mutants of specific LHC proteins will pinpoint the interaction partners of LHCSR1 and help us elucidate how PSBS and LHCSR1 evolved.

## Electronic supplementary material

Below is the link to the electronic supplementary material.

Supplementary material 1 (DOCX 8947 kb)
