# Supplementary material for: Functional analysis of LHCSR1, a protein catalyzing NPQ in mosses, by heterologous expression in Arabidopsis thaliana
Source: Photosynth Res. 2019 Jul 3;142(3):249–64. doi: 10.1007/s11120-019-00656-3 (PMC6874524; doi:10.1007/s11120-019-00656-3)
Supplement: Supplementary file 1 — Supplementary material 1 (DOCX 8947 kb) [file 11120_2019_656_MOESM1_ESM.docx]

**Supplementary data**

**Functional analysis of LHCSR1, a protein catalyzing NPQ in mosses, by heterologous expression in *Arabidopsis thaliana***

**Photosynthesis Research**

Ioannis Dikaios*^,1^, Christo Schiphorst*^,1^, Luca Dall’Osto^1^, Alessandro Alboresi^1^, Roberto Bassi^1^ and Alberta Pinnola^1,2,3^

*^1^ Department of Biotechnology, University of Verona, Verona, 37134, Italy*

*^2^ Department of Biology and Biotechnology, University of Pavia, Pavia, 27100, Italy*

^3^corresponding author

^*^equal contribution

[alberta.pinnola@unipv.it](mailto:alberta.pinnola@unipv.it)

|  | | **Fv/Fm** | | | |
| --- | --- | --- | --- | --- | --- |
|  |  | Cycle 1 | Cycle 2 | Cycle 3 | Cycle 4 |
| Fig. 3 | At WT | 0.873±0.007 | 0.830±0.005 | 0.807±0.005 | 0.798±0.008 |
|  | At *npq4* | 0.874±0.002 | 0.825±0.002 | 0.800±0.002 | 0.787±0.002 |
|  | At *npq4*+SR1 | 0.870±0.003 | 0.821±0.004 | 0.793±0.005 | 0.784±0.005 |
|  |  |  |  |  |  |
| Fig. 5 | Pp *psbs-lhcsr2 ko* | 0.839±0.006 | 0.726±0.010 | 0.743±0.004 | 0.722±0.011 |
|  | At WT | 0.873±0.007 | 0.830±0.005 | 0.807±0.005 | 0.798±0.008 |
|  | At *npq4* | 0.874±0.002 | 0.825±0.002 | 0.800±0.002 | 0.787±0.002 |
|  | At npq4+SR1 | 0.870±0.003 | 0.821±0.004 | 0.793±0.005 | 0.784±0.005 |
|  |  |  |  |  |  |
| Fig. 7 | *npq1npq4* | 0.855±0.004 | 0.818±0.005 | 0.799±0.006 | 0.789±0.010 |
|  | *npq1npq4* + SR1 L A1 | 0.854±0.003 | 0.816±0.007 | 0.800±0.012 | 0.787±0.009 |
|  | *npq1npq4* + SR1 L B1 | 0.854±0.007 | 0.814±0.004 | 0.793±0.015 | 0.776±0.015 |
|  | *npq1npq4* + SR1 L C3 | 0.852±0.005 | 0.814±0.007 | 0.794±0.008 | 0.777±0.008 |
|  |  |  |  |  |  |
|  | *npq2npq4* | 0.858±0.004 | 0.792±0.007 | 0.766±0.006 | 0.756±0.005 |
|  | *npq2npq4* + SR1 L C1 | 0.856±0.003 | 0.797±0.004 | 0.778±0.004 | 0.768±0.005 |
|  | *npq2npq4* + SR1 L C2 | 0.840±0.005 | 0.778±0.004 | 0.761±0.006 | 0.750±0.006 |
|  | *npq2npq4* + SR1 L C3 | 0.844±0.006 | 0.787±0.010 | 0.770±0.013 | 0.761±0.015 |
|  |  |  |  |  |  |
|  | *lut2npq4* | 0.863±0.003 | 0.824±0.005 | 0.791±0.004 | 0.773±0.006 |
|  | *lut2npq4* + SR1 L A1 | 0.855±0.003 | 0.822±0.001 | 0.789±0.006 | 0.775±0.004 |
|  | *lut2npq4* + SR1 L B1 | 0.854±0.003 | 0.816±0.003 | 0.782±0.002 | 0.771±0.004 |
|  | *lut2npq4* + SR1 L C1 | 0.852±0.002 | 0.818±0.004 | 0.788±0.003 | 0.775±0.006 |

**Table S1.**

The Fv/Fm values from the different cycles applied to leaves of the measured mutants and different LHCSR1-expressing lines. Standard deviation is reported behind the values.

|  |  | Fv/Fm |  |  |  | Fv/Fm |
| --- | --- | --- | --- | --- | --- | --- |
| 100uE | *npq4* | 0.775 ± 0.012 |  | 200uE | *npq4* | 0.771 ± 0.009 |
|  | WT | 0.790 ± 0.007 |  |  | WT | 0.791 ± 0.007 |
|  | *npq4*+SR1 L. C1 | 0.774 ± 0.005 |  |  | *npq4*+SR1 L. C1 | 0.772 ± 0.004 |
|  | *npq4*+SR1 L. A5 | 0.780 ± 0.017 |  |  | *npq4*+SR1 L. A5 | 0.771 ± 0.016 |
|  | npq4+SR1 L. A1 | 0.777 ± 0.004 |  |  | *npq4*+SR1 L. A1 | 0.769 ± 0.012 |
|  |  |  |  |  |  |  |
| 400uE | *npq4* | 0.778 ± 0.004 |  | 600uE | *npq4* | 0.772 ± 0.006 |
|  | WT | 0.792 ± 0.007 |  |  | WT | 0.789 ± 0.006 |
|  | *npq4*+SR1 L. C1 | 0.773 ± 0.002 |  |  | *npq4*+SR1 L. C1 | 0.772 ± 0.007 |
|  | *npq4*+SR1 L. A5 | 0.777 ± 0.007 |  |  | *npq4*+SR1 L. A5 | 0.769 ± 0.009 |
|  | *npq4*+SR1 L. A1 | 0.772 ± 0.010 |  |  | *npq4*+SR1 L. A1 | 0.771 ± 0.009 |
|  |  |  |  |  |  |  |
| 800uE | *npq4* | 0.772 ± 0.007 |  | 1000uE | *npq4* | 0.768 ± 0.011 |
|  | WT | 0.794 ± 0.006 |  |  | WT | 0.786 ± 0.004 |
|  | *npq4*+SR1 L. C1 | 0.771 ± 0.006 |  |  | *npq4*+SR1 L. C1 | 0.771 ± 0.008 |
|  | *npq4*+SR1 L. A5 | 0.772 ± 0.004 |  |  | *npq4*+SR1 L. A5 | 0.762 ± 0.004 |
|  | *npq4*+SR1 L. A1 | 0.775 ± 0.010 |  |  | *npq4*+SR1 L. A1 | 0.765 ± 0.015 |

**Table S2.**

Fv/Fm of *npq4*+LHCSR1 lines in various light intensities. Three different *A. thaliana npq4*+LHCSR1 lines with high and intermediate NPQ activation (line C1, A1 and A5) were tested in a variety of actinic light intensities. Leaves (n=3) were dark adapted for 45min, pre-treated with 800µmol photons∙m^-2^∙s^-1^ of actinic light for 15min and left to relax in the dark for 10min before the NPQ measurement. Each measurement corresponds to one single NPQ cycle of 5min different with different actinic light intensities and 5min dark recovery. The actinic light intensities used were: 100, 200, 400, 600, 800 and 1000µmol photons∙m^-2^∙s^-1^ (µE) from a-f respectively. Leaves from *A. thaliana* WT and *npq4* were used as control.

|  | **Chl *a/b*** | **Car/Chl** | **V+A+Z** | **DEP** | **Neo** | **Vio** | **Ant** | **Lut** | **Zea** | **β-car** |
| --- | --- | --- | --- | --- | --- | --- | --- | --- | --- | --- |
| T_0__Dark |  |  |  |  |  |  |  |  |  |  |
| *npq4* | 2.59 ± 0.08 | 26.4 ± 1.1 | 2.6 ± 0.2 |  | 4.0 ± 0.2 | 2.6 ± 0.2 |  | 12.5 ± 0.5 |  | 6.4 ± 0.7 |
| Line C1 | 2.65 ± 0.09 | 27.1 ± 1.4 | 3.3 ± 0.8 |  | 3.9 ± 0.1 | 3.3 ± 0.8 |  | 12.8 ± 0.5 |  | 6.2 ± 0.1 |
| Line C3 | 2.60 ± 0.05 | 28.5 ± 1.7 | 3.6 ± 0.8 |  | 4.2 ± 0.4 | 3.6 ± 0.8 |  | 13.7 ± 0.9 |  | 5.9 ± 0.6 |
| T_10__Light |  |  |  |  |  |  |  |  |  |  |
| *npq4* | 2.50 ± 0.07 | 26.6 ± 0.7 | 3.4 ± 0.1 | 0.53 ± 0.07 | 4.1 ± 0.2 | 1.4 ± 0.2 | 0.3 ± 0.1 | 12.8 ± 0.5 | 1.7 ± 0.3 | 6.3 ± 0.2 |
| Line C1 | 2.59 ± 0.13 | 26.7 ± 1.2 | 4.3 ± 0.1 | 0.44 ± 0.04 | 3.9 ± 0.3 | 2.2 ± 0.1 | 0.4 ± 0.0 | 12.9 ± 1.0 | 1.7 ± 0.2 | 5.7 ± 0.7 |
| Line C3 | 2.59 ± 0.03 | 28.1 ± 0.9 | 4.7 ± 0.2 | 0.48 ± 0.02 | 4.1 ± 0.1 | 2.2 ± 0.2 | 0.4 ± 0.0 | 13.4 ± 0.1 | 2.0 ± 0.1 | 5.8 ± 0.5 |
| T_20__Dark |  |  |  |  |  |  |  |  |  |  |
| *npq4* | 2.60 ± 0.09 | 26.7 ± 2.7 | 3.4 ± 0.2 | 0.53 ± 0.01 | 4.0 ± 0.7 | 1.3 ± 0.1 | 0.6 ± 0.0 | 12.6 ± 1.6 | 1.5 ± 0.1 | 6.7 ± 0.6 |
| Line C1 | 2.65 ± 0.08 | 26.4 ± 1.1 | 4.2 ± 0.4 | 0.42 ± 0.03 | 3.6 ± 0.1 | 2.1 ± 0.3 | 0.6 ± 0.1 | 12.6 ± 0.3 | 1.5 ± 0.1 | 6.0 ± 0.5 |
| Line C3 | 2.58 ± 0.06 | 28.1 ± 2.4 | 4.7 ± 1.2 | 0.48 ± 0.02 | 4.1 ± 0.4 | 2.2 ± 0.7 | 0.6 ± 0.1 | 13.6 ± 1.2 | 2.0 ± 0.4 | 5.7 ± 0.3 |
| T_30__Light |  |  |  |  |  |  |  |  |  |  |
| *npq4* | 2.48 ± 0.03 | 27.6 ± 1.4 | 3.6 ± 0.2 | 0.63 ± 0.01 | 4.3 ± 0.3 | 1.2 ± 0.1 | 0.3 ± 0.1 | 13.4 ± 0.6 | 2.1 ± 0.1 | 6.3 ± 0.6 |
| Line C1 | 2.63 ± 0.01 | 27.1 ± 2.0 | 4.4 ± 0.6 | 0.54 ± 0.03 | 3.8 ± 0.3 | 1.9 ± 0.4 | 0.4 ± 0.1 | 13.0 ± 1.0 | 2.2 ± 0.2 | 5.9 ± 0.1 |
| Line C3 | 2.60 ± 0.07 | 28.1 ± 1.2 | 4.4 ± 0.5 | 0.61 ± 0.01 | 4.1 ± 0.3 | 1.5 ± 0.2 | 0.4 ± 0.0 | 13.5 ± 0.9 | 2.5 ± 0.3 | 6.2 ± 0.2 |
| T_40__Dark |  |  |  |  |  |  |  |  |  |  |
| *npq4* | 2.53 ± 0.03 | 27.6 ± 0.9 | 3.6 ± 0.2 | 0.59 ± 0.02 | 4.2 ± 0.2 | 1.2 ± 0.1 | 0.6 ± 0.1 | 13.2 ± 0.4 | 1.8 ± 0.2 | 6.6 ± 0.2 |
| Line C1 | 2.55 ± 0.06 | 26.5 ± 1.1 | 4.0 ± 0.6 | 0.51 ± 0.02 | 3.8 ± 0.1 | 1.6 ± 0.3 | 0.7 ± 0.1 | 13.1 ± 0.8 | 1.7 ± 0.2 | 5.7 ± 0.6 |
| Line C3 | 2.54 ± 0.09 | 27.6 ± 0.8 | 4.1 ± 0.5 | 0.56 ± 0.03 | 4.1 ± 0.1 | 1.5 ± 0.3 | 0.7 ± 0.0 | 13.4 ± 0.8 | 2.0 ± 0.3 | 6.1 ± 0.4 |

**Table S3.**

HPLC-pigment analysis of *A. thaliana npq4* and two lines expressing LHCSR1 (line C1 and C3). Dark adapted samples were analyzed as well as samples harvested at the end of alternated light/dark cycles of 10 minutes (light intensity: 1200µmol photons∙m^-2^∙s^-1^). Pigments are normalized to 100 Chl molecules and analysis was performed in triplicates. The DEP was calculated by the following formula: (Zea+0.5*Ant)/(Zea+Vio+Ant).


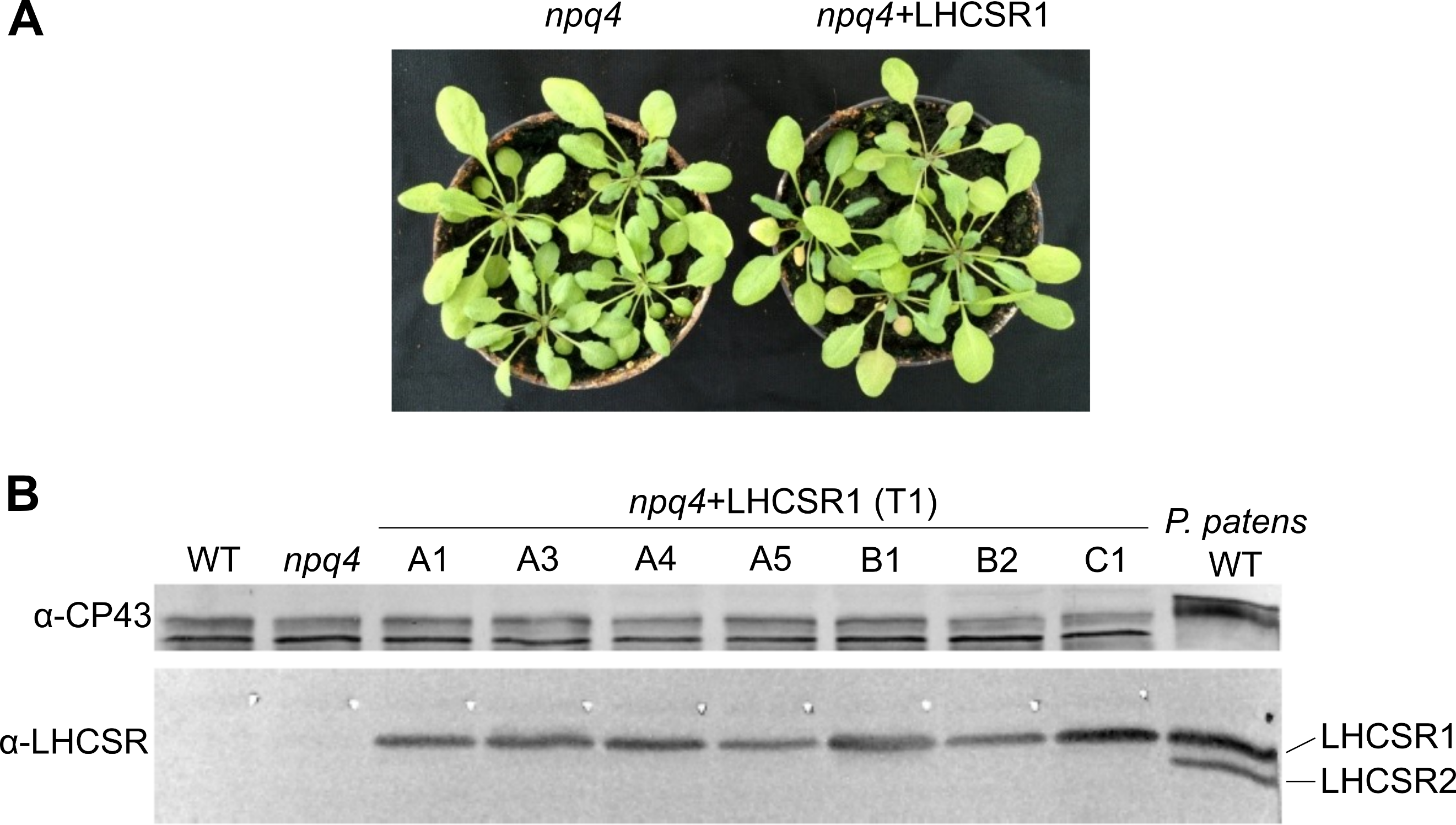


**Figure S1**.

Biochemical characterization of the *A. thaliana* *npq4* lines transformed with LHCSR1. The presence of the LHCSR1 subunit was assessed by Western blot. (A) Different lines are shown after 5 weeks of growth. (B) Western blot analysis was performed on total proteins extracted by grinding one leaf disk directly in 100 µL of loading buffer, one tenth of the volume was loaded on an SDS-PAGE. Proteins of WT and *npq4* plants were loaded as a control as well as the equivalent of 1 µg of Chl of thylakoids from WT *P. patens* plants. The primary antibody used for the analysis is indicated on the left side of the membrane while the band corresponding either to *P. patens* LHCSR1 or LHCSR2 is indicated on the right side.


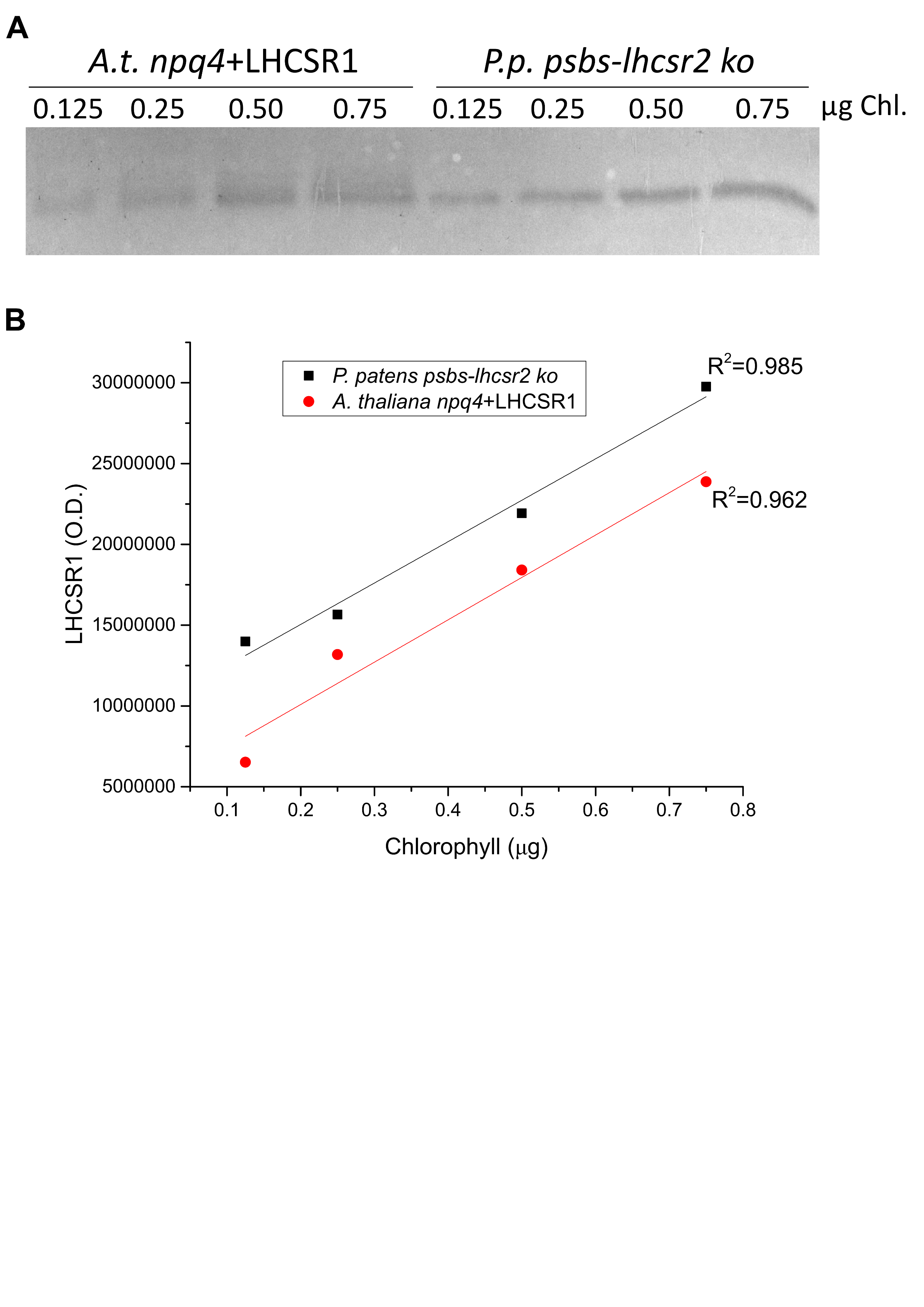


**Figure S2**.

Comparison of LHCSR1 content in *P. patens psbs-lhcsr2 ko* and *A. thaliana npq4*+LHCSR1. (A) Different concentrations of Chl from whole leaf extracts of *A. thaliana npq4*+LHCSR1 or tissue of *P. patens* *psbs-lhcsr2ko* were loaded on an SDS-PAGE and blotted against the α-LHCSR antibody. (B) The O.D. of LHCSR1 determined from the western blot was plotted against the Chl concentrations to determine the amount of LHCSR1 in *P. patens* and *A. thaliana*.


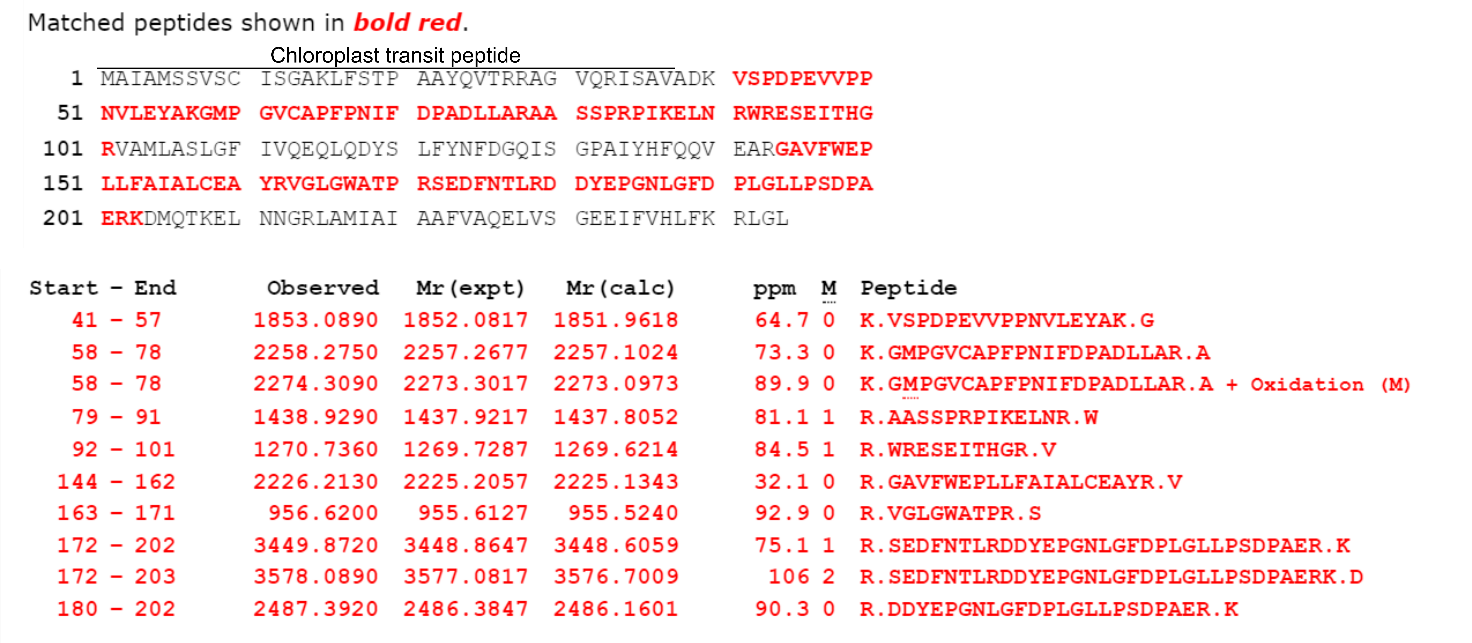


**Figure S3.**

The band from LHCSR1 expressed in *A. thaliana* thylakoids was excised. The protein sample was digested with trypsin overnight. The peptides obtained were analyzed using a matrix-assisted laser desorption ionization time-of-flight mass spectrometer (KRATOS Analytical, Shimadzu corporation, Japan). Database search was performed by the Mascot wizard from www.matrixscience.com. The protein identification results were obtained from the primary mass spectrum of the peptides produced after enzymatic hydrolysis. Search parameters: Trypsin enzymatic solution, set two missed cut sites. The alkylation of cysteine is set to a fixed modification, and the oxidation of methionine as a variable modification. The database used for the authentication was NCBInr.


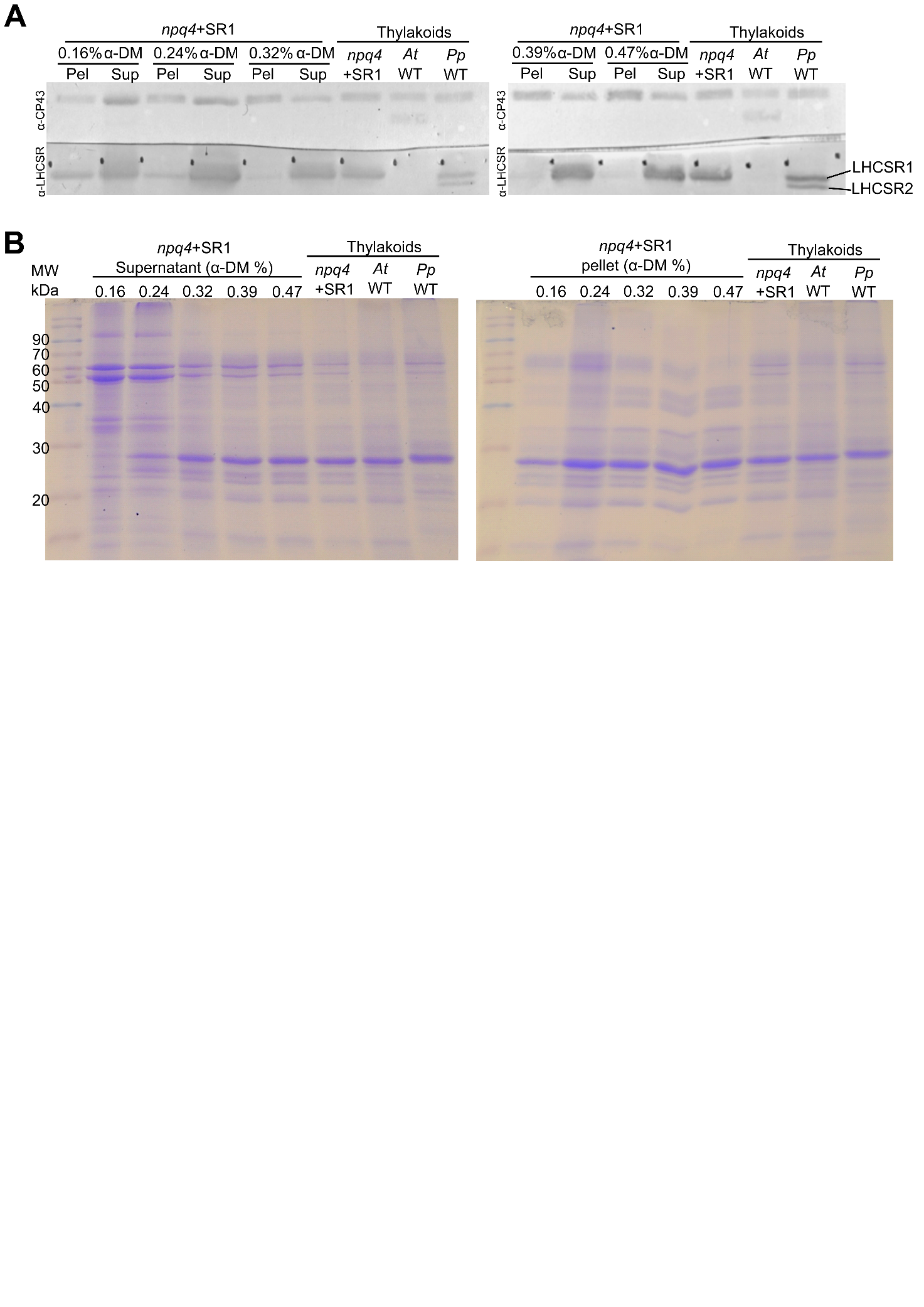


**Figure S4.**

Grana and stroma separation from *npq4*+LHCSR1 thylakoids by fractionation with different α-DM concentrations*.* (A) Western blot analysis of LHCSR1 distribution of the pellet and supernatant upon fractionation of isolated thylakoids with different concentrations of α-DM (0.16-0.47%). The pellet (Pel) is enriched in the grana fractions, while the supernatant (Sup) mostly contains stroma fractions. Fractions and thylakoids were loaded on SDS-PAGE gels. Equal amounts of thylakoids from *A. thaliana npq4*+LHCSR1 (*npq4*+SR1), *A. thaliana* WT (*At* WT) and *P. patens* WT (*Pp* WT) were loaded as controls. All samples were loaded on a Chl basis of 0.5µg. (B) Coomassie staining of thylakoids and fractionated thylakoid membranes, samples were loaded on a Chl basis of 2µg.


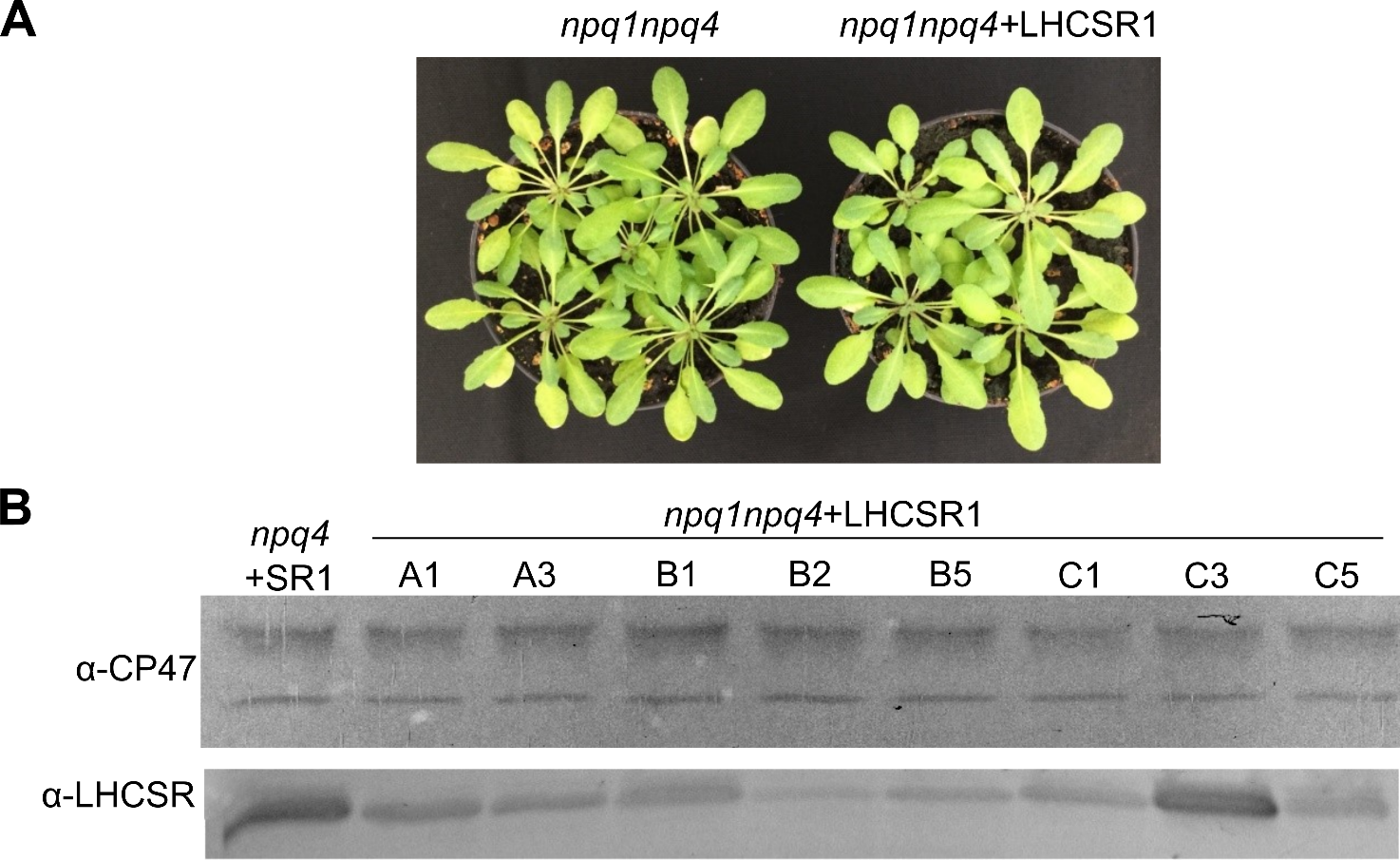


**Figure S5**.

Immunological screening of *npq1npq4*+LHCSR1 transformed lines. (A) *A. thaliana* transgenic lines were selected on agar plates and then transferred in pots in a short-day photoperiod growth chamber (right). Control *npq1npq4* plants of the same age were also grown in the same conditions (left). (B)Western blot analysis of 8 independent complemented lines from the T2 generation and *npq4*+LHCSR1 as a control.


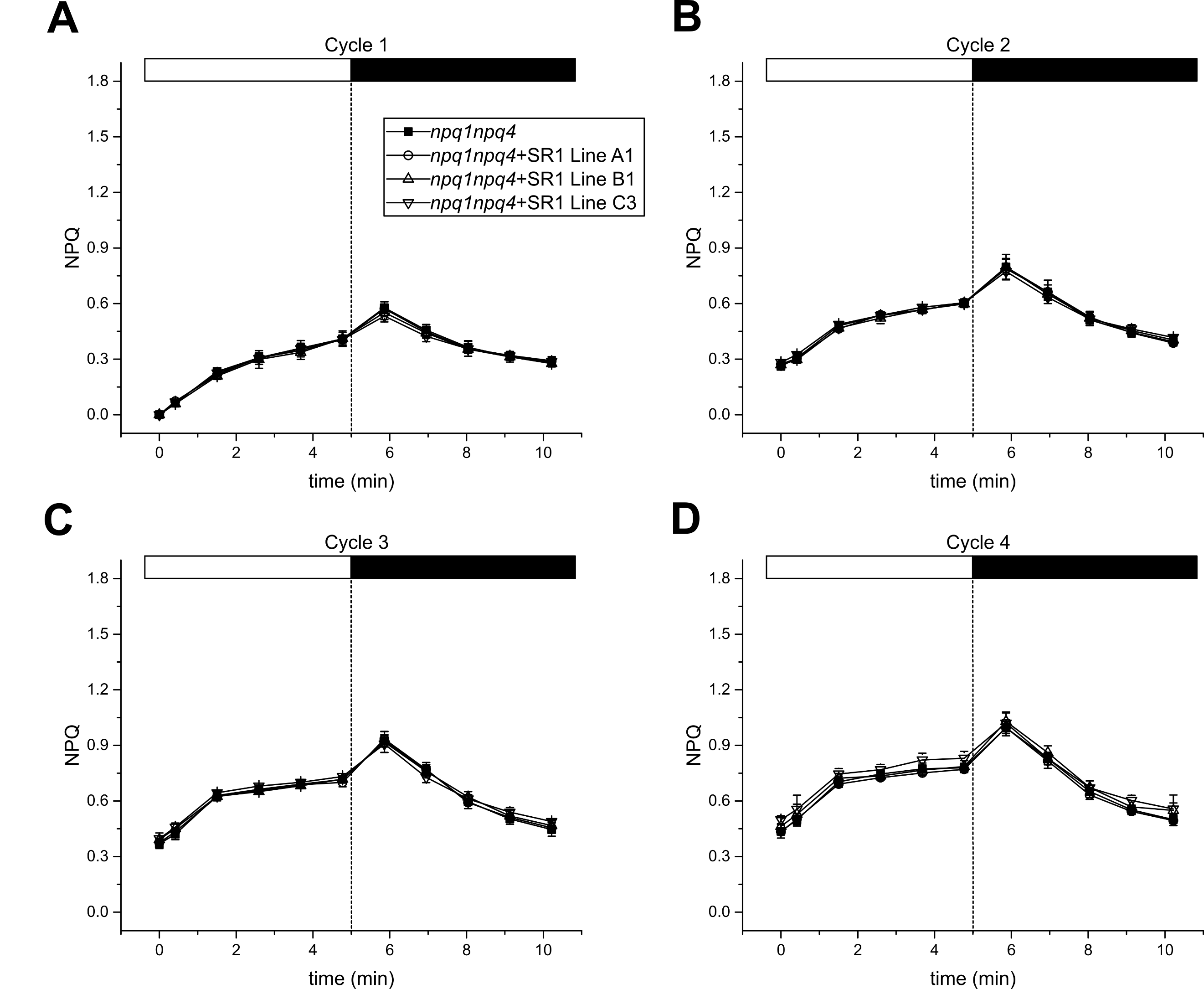


**Figure S6.**

NPQ measurements in *A. thaliana npq1npq4* complemented with LHCSR1 using fluorescence video-imaging (n=3). NPQ of Chl fluorescence was measured in leaves taken from 4-5-week old plants. Four successive NPQ cycles were measured, protocol: 5 minutes of actinic light treatment (1200µmol photons∙m^-2^∙s^-1^) followed by 5 min of dark recovery. The four cycles are presented by A-D respectively.


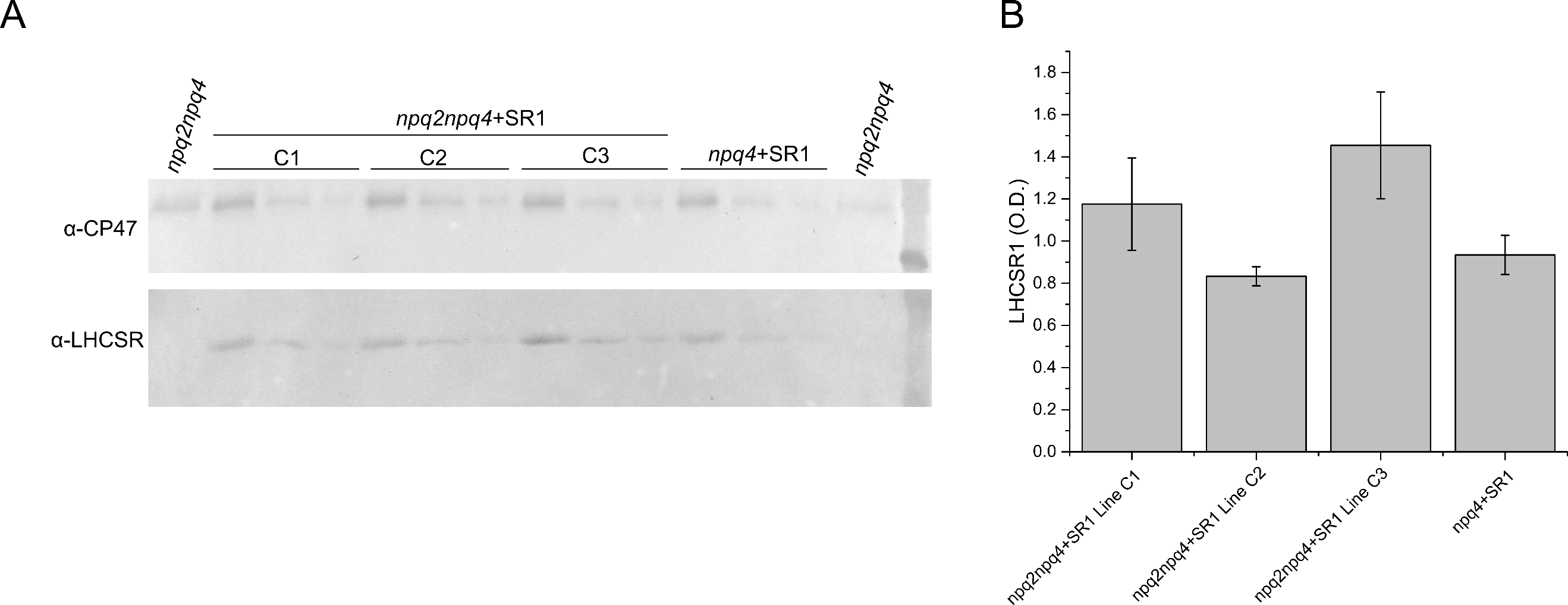


**Figure S7**.

Immunological screening and immuno-titration of *npq2npq4*+LHCSR1 transformed lines. (A) Western blot analysis of was performed on total protein extracts. Chl concentration was determined, and different amounts were loaded on the gel. Proteins of non-transformed (*npq2npq4*) and transgenic *npq4*+LHCSR1 plants were loaded as controls. (B) The O.D. of LHCSR1 was determined from the western blot was plotted against the Chl concentrations to determine the amount of LHCSR1 in the *npq2npq4* complemented lines in comparison to *npq4*+LHCSR1.


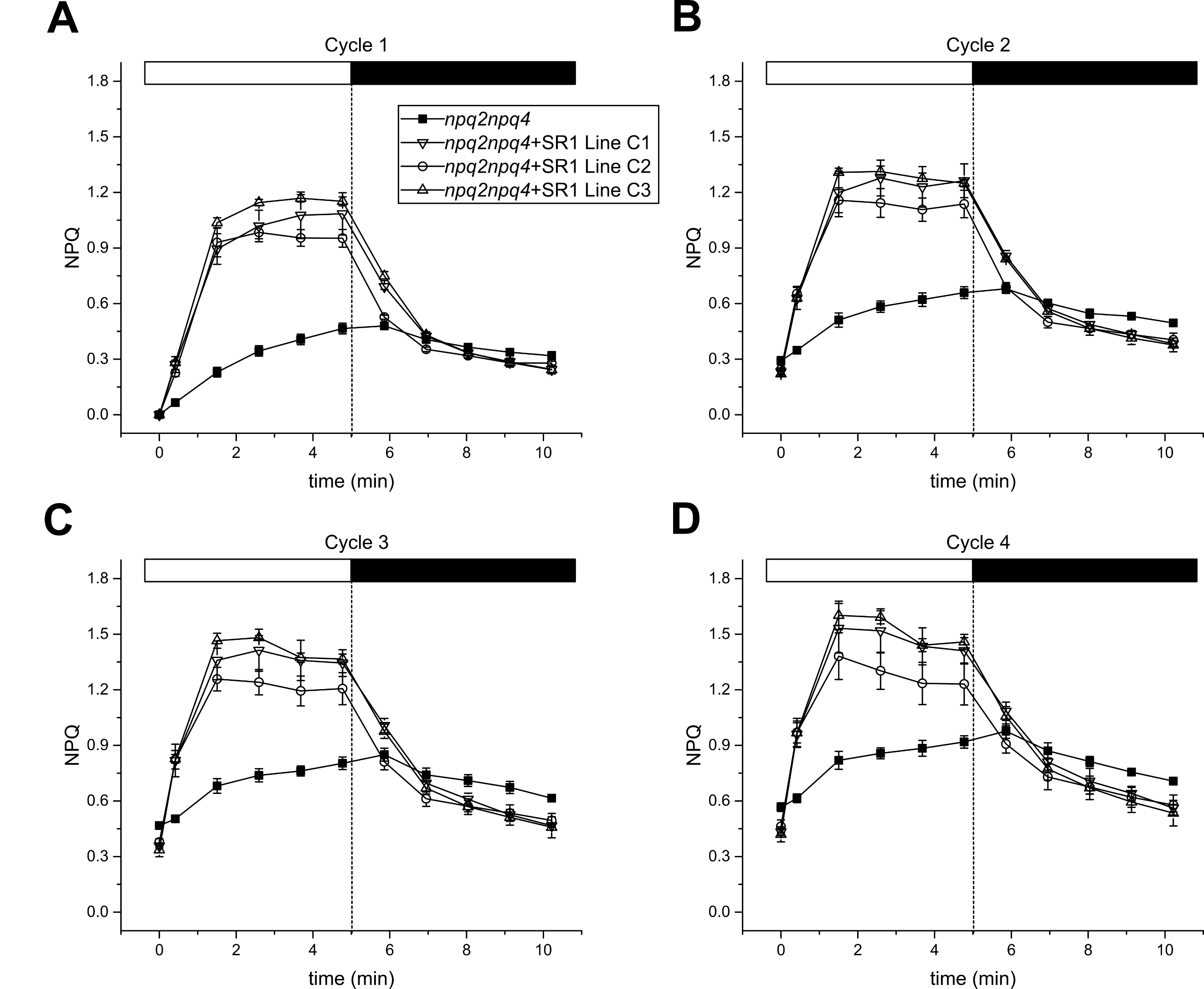


**Figure S8.**

NPQ measurements in different *A. thaliana npq2npq4* complemented with LHCSR1 using fluorescence video-imaging (n=3). NPQ of Chl fluorescence was measured in leaves taken from 4-5-week old *npq2npq4* and 3 complemented lines. Four successive NPQ cycles were measured; 5 minutes of actinic light treatment (1200µmol photons∙m^-2^∙s^-1^) followed by 5 min of dark recovery. The four cycles are presented by A-D respectively.


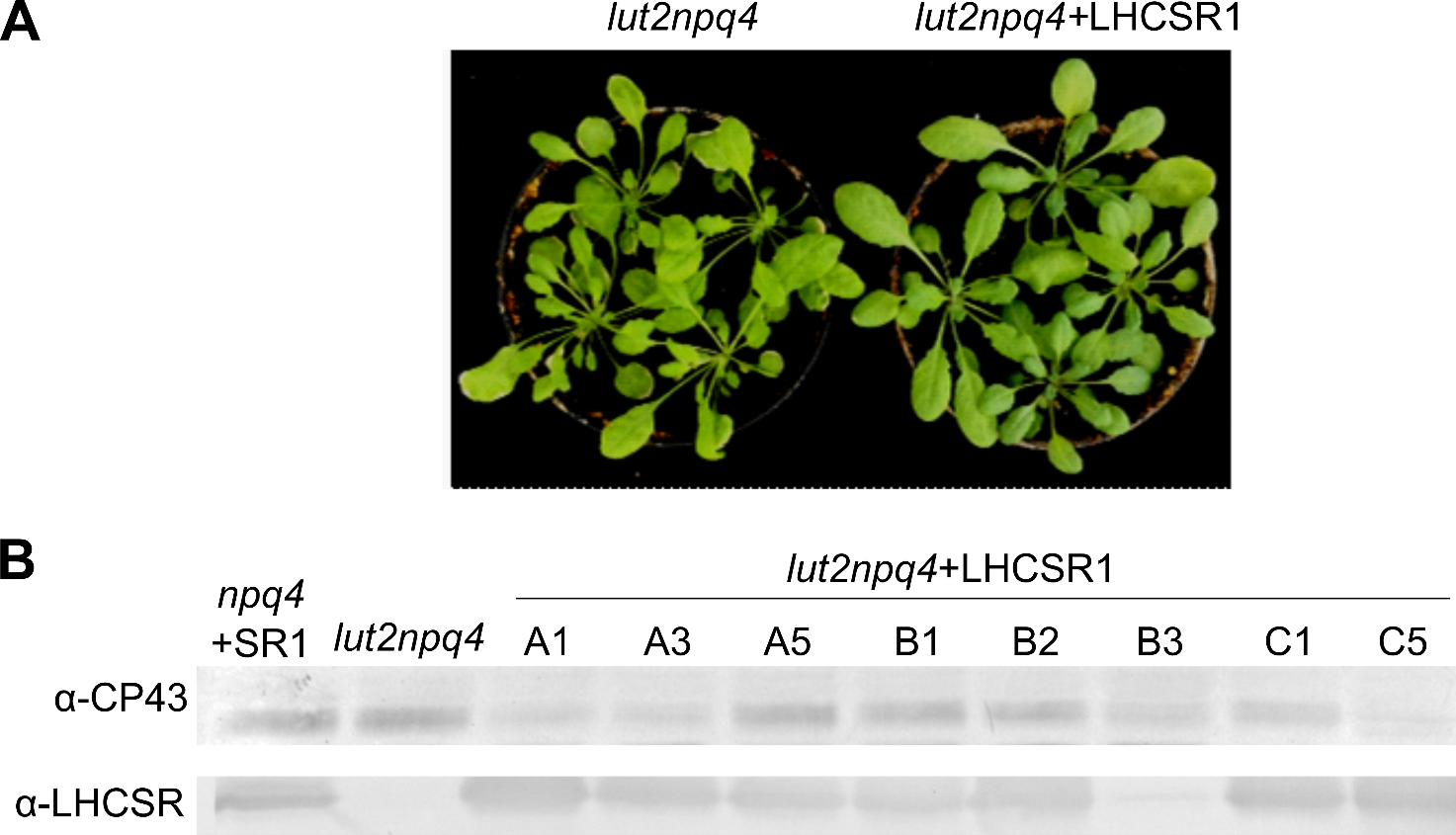


**Figure S9**.

Immunological screening of *lut2npq4*+LHCSR1 transformed lines (n=3). (A) *A. thaliana* transgenic lines were selected on agar plates supplemented with hygromycin-B. Control *lut2npq4* plants of the same age were also grown in the same conditions. (B)Western blot analysis of 8 independent plant lines together with *lut2npq4* and *npq4*+LHCSR1 as controls.


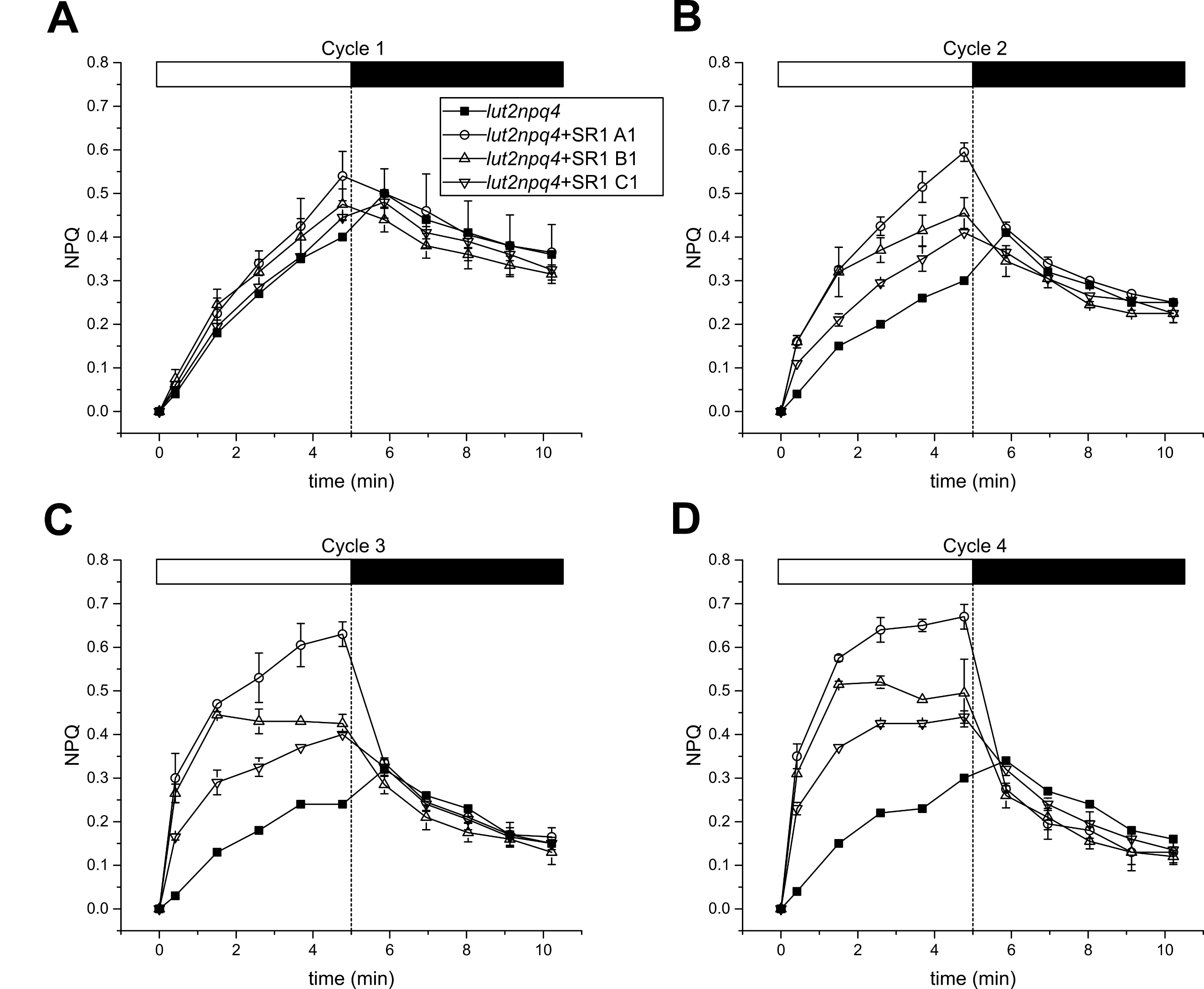


**Figure S10.**

NPQ measurements in different *A. thaliana lut2npq4* complemented with LHCSR1 using fluorescence video-imaging (n=3). NPQ of Chl fluorescence was measured in leaves taken from 4-5-week old plants. Four successive NPQ cycles were measured, protocol: 5 minutes of actinic light treatment (1200µmol photons∙m^-2^∙s^-1^) followed by 5 minutes of dark recovery. The four cycles are presented by A-D respectively.


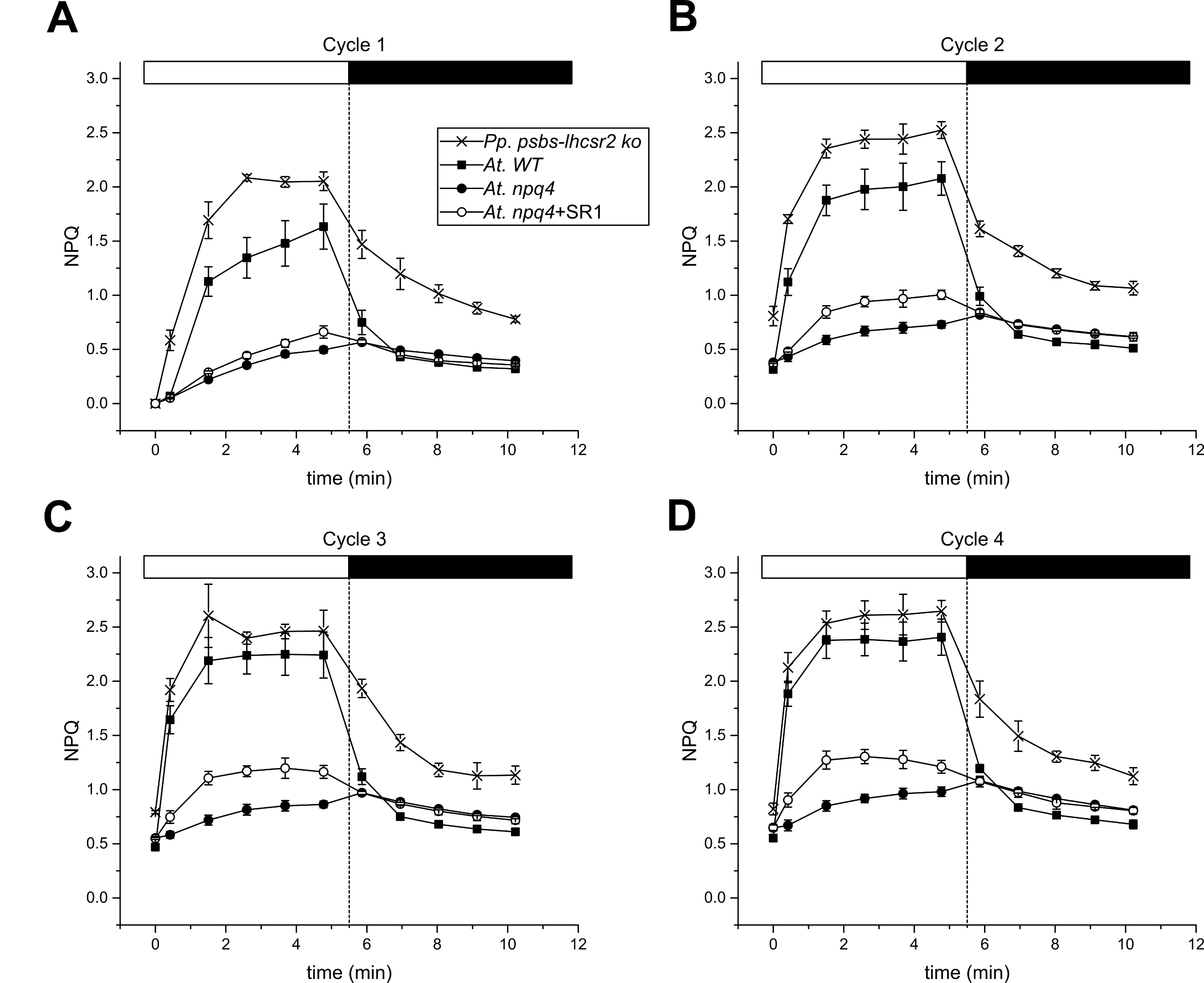


**Figure S11**.

NPQ measurements (n=4) in *A. thaliana WT, npq4, npq4*+LHCSR1 and *P. patens psbs-lhcsr2 ko (n=3)*. Measurements were performed with video-imaging at 1200µmol photons∙m^-2^∙s^-1^ with 4 cycles of 5min light and 5 min dark, figure A-D respectively.


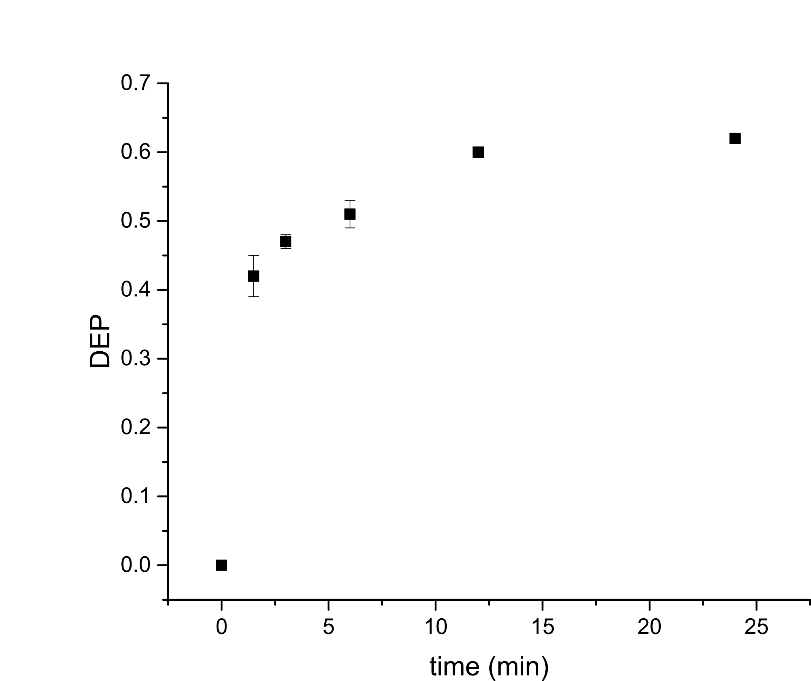


**Figure S12**.

Time course of the Deepoxidation index (DEP) in *P. patens* (n=3). Plants were subjected to 850µmolphotons∙m^-2^∙s^-1^ and samples were taken and frozen in liquid nitrogen after different time intervals to be analyzed by HPLC. The DEP was calculated by the following formula: (Zea+0.5*Ant)/(Zea+Vio+Ant).
